# Coronavirus replicase epitopes induce cross-reactive CD8 T cell responses in SARS-CoV-2-naive people with HIV-1

**DOI:** 10.1016/j.isci.2025.111949

**Published:** 2025-02-03

**Authors:** Katja G. Schmidt, Paulina Geißler, Ev-Marie Schuster, Christine Schülein, Ellen G. Harrer, Verena Schönau, Markus Luber, Bernd Spriewald, Philipp Steininger, Silke Bergmann, Armin Ensser, Kilian Schober, Krystelle Nganou-Makamdop, Thomas Harrer

**Affiliations:** 1 Infectious Diseases and Immunodeficiency Section, Department of Medicine 3, Rheumatology and Immunology, Friedrich-Alexander-Universität Erlangen-Nürnberg and Uniklinikum Erlangen, Erlangen, Germany; 2 Mikrobiologisches Institut – Klinische Mikrobiologie, Immunologie und Hygiene, Friedrich-Alexander-Universität Erlangen-Nürnberg and Uniklinikum Erlangen, Erlangen, Germany; 3 Department of Medicine 3, Rheumatology and Immunology, Friedrich-Alexander-Universität Erlangen-Nürnberg and Uniklinikum Erlangen, Erlangen, Germany; 4 Department of Medicine 5, Friedrich-Alexander-Universität Erlangen-Nürnberg and Uniklinikum Erlangen, Erlangen, Germany; 5 FAU Profile Center Immunomedicine, Friedrich-Alexander-Universität Erlangen-Nürnberg (FAU) Erlangen-Nürnberg, Erlangen, Germany; 6 Institute of Clinical and Molecular Virology, Friedrich-Alexander-Universität Erlangen-Nürnberg and Uniklinikum Erlangen, Erlangen, Germany

**Keywords:** Health sciences, Medicine, Medical specialty, Immunology, Virology

## Abstract

Cross-reactive T cell immunity between common cold coronaviruses and SARS-CoV-2 may influence COVID-19 susceptibility. To identify cross-reactive CD8 T cell epitopes, we analyzed responses to 21 homologous SARS-CoV-2 replicase peptides in 177 people living with HIV (PLWH) on antiretroviral therapy, of which 133 did not have prior SARS-CoV-2 infection. Replicase peptides induced IFN-γ responses in 63% of the SARS-CoV-2-naïve individuals and in 73% of individuals with prior SARS-CoV-2-infection. We could define several cross-reactive epitopes, including the HLA-B∗35:03 restricted CoV-YL8, and characterized a CoV-YL8-specific T cell receptor cloned from a SARS-CoV-2 seronegative individual. Analysis of the association between HLA-I alleles and SARS-CoV-2 infections over a 16-months period revealed that in a cohort of 452 PLWH, HLA-B∗35:03 and C∗07 were underrepresented in the 55 persons with a history of SARS-CoV-2 infection while HLA-B∗35:01 and HLA-C∗04 were associated with a higher infection rate. Taken together, our study suggests an HLA-I-mediated effect of common cold coronaviruses on SARS-CoV-2 immunity.

## Highlights

• SARS-CoV-2 replicase cross-reactive T cells are frequent in SARS-CoV-2 naive PLWH
• A functional TCR for the B∗35:03-restricted replicase epitope YL8 was identified
• While HLA-B∗35 and C∗04 increase SARS-CoV-2 susceptibility, C∗07 is protective
• HLA-B∗35:01 and 35:03 subtypes have diverging effects on SARS-CoV-2 susceptibility

## Graphical abstract

**Figure:**
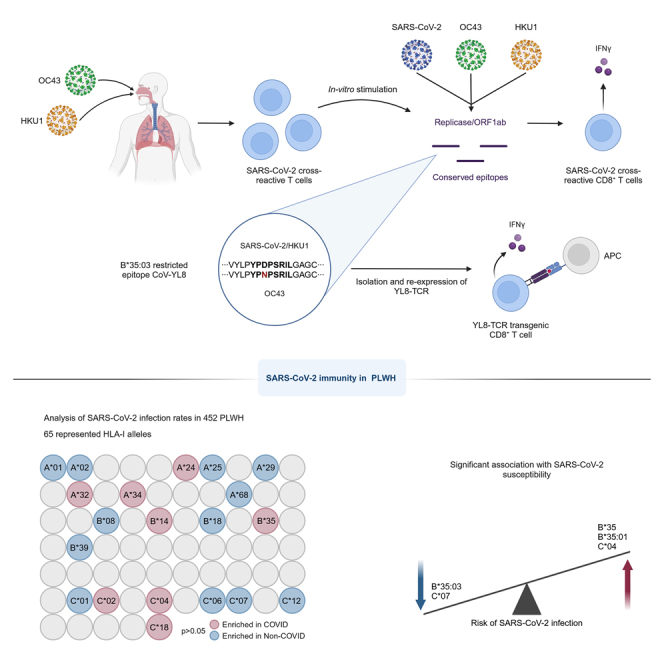

## Introduction

The rapid development of effective vaccines played an important role for the control of the SARS-CoV-2 pandemic. Aside from inactivated whole virus vaccines, current SARS-CoV-2 vaccines focus on eliciting immune responses against the viral spike protein.^1^^,^^2^^,^^3^^,^^4^^,^^5^ However, even variant adapted vaccines have a limited efficacy for protection from infection due to the rapid emergence of variants evading neutralization by vaccine-induced antibodies.^6^^,^^7^ Given the growing evidence that SARS-CoV-2 specific T cell responses are important for the protection from SARS-CoV-2 infection and severe disease,^8^^,^^9^^,^^10^^,^^11^ the inclusion of T cell epitopes derived from viral proteins other than spike could enhance vaccine efficacy. Moreover, T cell cross-reactivity induced by epitopes derived from conserved genomic regions between SARS-CoV-2 and common cold coronaviruses (CCCoVs) has inspired the concept of a potentially more effective pan-coronavirus vaccine. So far, CCCoV/SARS-CoV-2 cross-reactive T cells have been reported for epitopes in the SARS-CoV-2 nucleocapsid,^12^^,^^13^^,^^14^^,^^15^^,^^16^ spike,^12^^,^^13^^,^^17^ envelope,^13^ matrix,^12^ and several non-structural proteins.^12^^,^^13^ However, these studies have almost exclusively been conducted with healthy donors and did not include people living with HIV (PLWH). Even under effective antiretroviral therapy, the numerous perturbations of the T cell compartment in PLWH include altered T cell subset frequencies, T cell exhaustion, lower levels of CD4 T cell counts, decreased T cell receptor (TCR) repertoire diversity, alterations in the use of V segments and skewed CDR3 length distribution.^18^^,^^19^^,^^20^^,^^21^^,^^22^^,^^23^^,^^24^ Thus, pan-coronavirus candidate epitopes identified in people living without HIV (PLWOH) may not be optimal targets for people with HIV. Here, we sought to identify cross-reactive T cell epitopes specifically in PLWH using the SARS-CoV-2 replicase polyprotein—a highly conserved genomic region among coronaviruses—as key target. To this end, we analyzed T cell responses induced by a set of SARS-CoV-2 replicase peptides that have high homology to the CCCoVs OC43 and HKU1 sequences and assessed replicase specific T cell responses in PLWH with or without prior SARS-CoV-2 infection.

## Results

### Individuals without prior infection respond to SARS-CoV-2 replicase peptides

To investigate the recognition of conserved epitopes within the SARS-CoV-2 replicase polyprotein, 21 peptides with a length of 11–22 amino acids and >73% homology to the replicase of the CCCoVs OC43 and HKU1 (Table S1) were tested in four pools of five to six peptides each. Recognition of these peptide pools across a cohort of 177 HIV-1 individuals was tested using peptide stimulation assays that are highly sensitive for the detection of functionally active antigen-specific T cells with low frequencies. While there was no indication of prior SARS-CoV-2 infection in the majority (75%) of the 177 individuals (non-COVID group), 44 of the 177 (25%) individuals (COVID group) had already experienced a prior SARS-CoV-2 infection as defined by presence of nucleocapsid antibodies or spike antibodies without prior vaccination.

**Figure 1:**
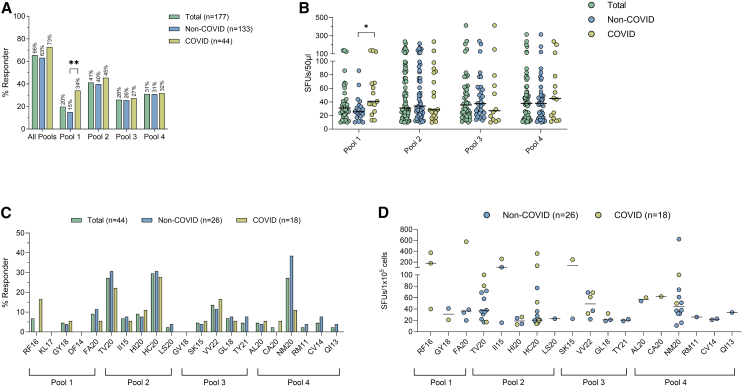
Recognition of conserved SARS-CoV-2 replicase peptides by stimulated T cell lines of individuals with or without prior SARS-CoV-2 infection (A) Percentage of 177 PLWH recognizing different replicase pools in IFN-γ ELISpot assay. For statistical comparisons, two-tailed Fisher’s exact tests were used. (B) Magnitude of IFN-γ response to the different replicase pools in the responders as defined by spot forming units (SFUs) after subtraction of background. The median number of SFUs in responders is indicated by the solid line and non-responders are not included. *n* responders: Pool 1: total = 35, non-COVID = 20, COVID = 15. Pool 2: total = 73, non-COVID = 53, COVID = 20. Pool 3: total = 46, non-COVID = 34, COVID = 12. Pool 4: total = 55, non-COVID = 41, COVID = 14. For statistical comparisons, two-tailed Mann-Whitney tests were used. (C) Percentage of individuals responding to individual peptides contained within the four peptide pools. For statistical comparisons, two-tailed Fisher’s exact tests were used. (D) Magnitude of IFN-γ response to the different peptides contained in the replicase pools as defined by spot forming units (SFUs) after subtraction of background. The median SFUs in responders is indicated by the solid line and non-responders are not included. For statistical comparisons, two-tailed Mann-Whitney tests were used. COVID: individuals with prior SARS-CoV-2 infection. Non-COVID: individuals without prior SARS-CoV-2 infection. ∗ = p ≤ 0.05, ∗∗ = p ≤ 0.01.

Overall, 116 of 177 (66%) screened individuals responded to at least one peptide pool in IFN-γ ELISpot assays (Figure 1A). An IFN-γ response to at least one peptide pool was observed in 84 of the 133 (63%) SARS-CoV-2 seronegative individuals and in 32 of the 44 (73%) individuals with a prior SARS-CoV-2 infection. Among the 177 individuals, the percentage of responders varied between peptide pools with the highest frequency observed for pool 2 (42%) and the lowest for pool 1 (20%), suggesting differences in the antigenicity of the tested peptides. Individuals with prior SARS-CoV-2 infection recognized peptide pool 1 more frequently (Figure 1A; *p* = 0.0087) and with a higher magnitude (Figure 1B; *p* = 0.0139) in comparison to individuals without prior SARS-CoV-2 infection. Pools 2, 3, and 4 showed no significant differences in the recognition frequency or magnitude of response between individuals with and without prior SARS-CoV-2 infection.

Assessment of responses to the 21 individual peptides contained in the four pools could be performed in 44 ongoing T cell lines and identified 14 peptides that induced responses in persons without prior SARS-CoV-2 infection, with three peptides—CoV-TV20, CoV-HC20, and CoV-NM20—inducing the highest number of responders (Figures 1C and 1D). Of note, there was no difference in the CD4 and CD8 T cell counts, CD4:CD8 T cell ratios, viral loads, age, and sex distribution between responders and non-responders as well as between the COVID and non-COVID groups (Figure S1).

### Several CD8 T cell epitopes can be found in SARS-CoV-2 replicase peptides

To identify the T cell epitopes within the recognized replicase peptides, we next performed mapping of epitopes targeted by those T cell lines that expanded *in vitro* to high enough cell numbers necessary for further analyses. These T cell lines recognized the peptides CoV-NM20, CoV-CA20, CoV-SK15, CoV-RF16, and CoV-VV22, which exhibited various degrees of reactivity among the COVID and non-COVID groups.

**Figure 2:**
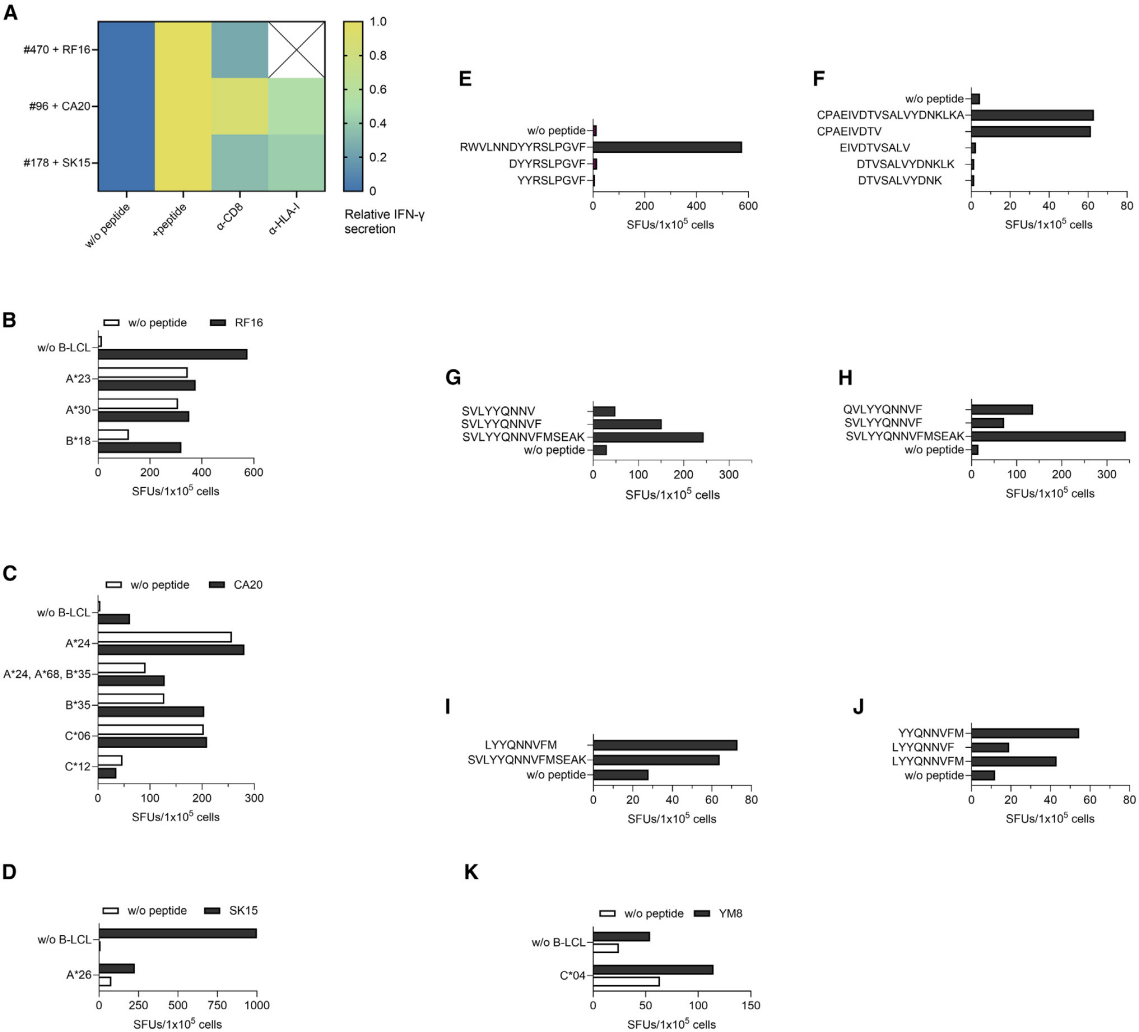
Recognition of replicase peptides by previously SARS-CoV-2 infected individuals (A) Blocking experiments with antibodies against CD8 and HLA-I in an IFN-γ ELISpot assay after stimulation of the T cell lines from donors #470, #96 and #178 with their respective peptides. IFN-γ secretion was normalized to the negative control (0) and the T cell line + peptide (1). (B–D) HLA-restriction analysis of the peptides (B) CoV-RF16 (#470), (C) CoV-CA20 (#96), and (D) CoV-SK15 (#178). The T cell lines were co-incubated with peptide-loaded B-LCLs in an IFN-γ ELISpot. Co-incubation with B-LCLs without peptide as well as direct incubation of T cells with the peptide without B-LCLs (w/o B-LCLs) served as a control. Shown are means of duplicates. (E–J) Recognition of shorter peptides within (E) CoV-RF16 (#470), (F) CoV-CA20 (#96), (G–I), and (J) CoV-SK15 (#178) in an IFN-γ ELISpot by the respective T cell line. CoV-RF16: RWVLNNDYYRSLPGVF, CoV-DF10: DYYRSLPGVF, CoV-YF9: YYRSLPGVF, CoV-CA20: CPAEIVDTVSALVYDNKLKA, CoV-CV9: CPAEIVDTV, CoV-EV10: EIVDTVSALV, CoV-DK13: DTVSALVYDNKLK, CoV-DK11: DTVSALVYDNK, CoV-SK15: SVLYYQNNVFMSEAK, CoV-SF10: SVLYYQNNVF, CoV-SV9: SVLYYQNNV, CoV-QF10: QVLYYQNNVF, CoV-LM9: LYYQNNVFM, CoV-YM8: YYQNNVFM, CoV-LF8: LYYQNNVF. (K) HLA-restriction analysis of the peptide CoV-YM8. The CoV-LM9 specific T cell line from individual #178 was co-incubated in an IFN-γ ELISpot with a CoV-YM8 loaded B-LCL sharing only HLA-C∗04^+^. Co-incubation with B-LCLs without peptide as well as direct incubation of T cells with the peptide without B-LCLs (w/o B-LCLs) served as a control. Shown are means of duplicates. SFUs: spot forming units. The prefix CoV- is omitted from the peptide names in the figure panels due to space restrictions.

We first performed epitope mapping in individuals with prior SARS-CoV-2 infection and identified three individuals who showed strong IFN-γ responses to single peptides. The T cell line from individual #470 responded to CoV-RF16, #96 showed a response to CoV-CA20 and #178 reacted to CoV-SK15. The IFN-γ responses toward all three peptides were markedly reduced upon blockade with anti-CD8 and anti-HLA-I antibodies, suggesting the presence of HLA-I restricted epitopes within those longer peptides (Figure 2A). Therefore, we co-incubated the T cell lines with peptide-loaded EBV-transformed B-lymphoblastoid cell lines (B-LCLs) sharing distinct HLA-I alleles of the respective individual. This revealed that individual #470 recognized an HLA-B∗18 restricted epitope within CoV-RF16 (Figure 2B), while the epitope in CoV-CA20 targeted by individual #96 was restricted to HLA-B∗35 (Figure 2C). Furthermore, we found that the T cell line from individual #178 recognized an HLA-A∗26-restricted epitope within CoV-SK15 (Figure 2D). To determine the actual epitope within those longer peptides, we first performed bioinformatic epitope prediction using the databases IEDB and Syfpeithi. The highest scores for a B∗18-restricted epitope within CoV-RF16 were obtained for the peptides CoV-DF10 (DYYRSLPGVF) and CoV-YF9 (YYRSLPGVF). However, no recognition toward either peptide was detected (Figure 2E); thus, the actual HLA-B∗18 restricted epitope within CoV-RF16 remains to be determined. While CoV-RF16 is completely conserved in HKU1 and shows 88% sequence homology to OC43, the homology to the two alpha-coronaviruses NL63 and 229E is only 25% and 19%, respectively. However, CoV-RF16 is conserved in all SARS-CoV-2 variants from Alpha to the current Omicron variants (BA.2.86, JN.1, KP.2, and KP.3).

Bioinformatic analysis predicted the peptide CoV-CV9 (CPAEIVDTV) as an HLA-B∗35-restricted epitope^25^^,^^26^ within the CoV-CA20 sequence, which was confirmed in a subsequent experiment using truncated peptides (Figure 2F). In stimulation experiments with an additional 48 B∗35 individuals, 7 of 48 (15%) responded to the peptide CoV-CV9 with 4/27 (15%) B∗35:01^+^, 3/11 (27%) B∗35:03^+^ and one out of four (25%) individuals with unknown B∗35 subtypes recognizing CoV-CV9 (Table S2). Other B∗35 subtypes (*n* = 6) did not react to CoV-CV9, possibly as those subtypes were underrepresented in our study. Five of the seven CoV-CV9 responders had prior SARS-CoV-2 infections, whereas two responders, including the responder with the highest IFN-γ secretion, did not have a prior SARS-CoV-2 infection, indicating that IFN-γ responses to the HLA-B∗35-restricted CoV-CV9 may be found in HLA-B∗35 individuals without prior SARS-CoV-2 infection. The epitope CoV-CV9 is conserved to 89% in OC43 and 78% in HKU1, the sequence identity to the alpha-coronaviruses NL63 and 229E is 89%. CoV-CV9 is also completely conserved in all SARS-CoV-2 variants from Alpha to the current Omicron variants (BA.2.86, JN.1, KP.2, and KP.3).

The databases IEDB and Syfpeithi predicted the peptide CoV-SF10 (SVLYYQNNVF) as an HLA-A∗26-restricted epitope within CoV-SK15. In experiments with shorter peptides, we indeed saw a response to CoV-SF10 by the T cell line from individual #178 and recognition was abrogated upon omission of the C-terminal phenylalanine (Figure 2G). In the CCCoVs OC43 and HKU1, the epitope is conserved with only a serine to glutamine substitution at position 1. Interestingly, this corresponding OC43/HKU1 peptide CoV-QF10 (QVLYYQNNVF) was recognized even better than CoV-SF10 (Figure 2H). The homology of CoV-SF10 is 90% to OC43 and HKU1, it is 80% conserved in NL63 and has a 70% homology to the 229E sequence. Moreover, CoV-SF10 is conserved in all SARS-CoV-2 variants from Alpha to the current Omicron variants (BA.2.86, JN.1, KP.2, and KP.3). As seen in Figure 2D, the response of the CoV-SK15-stimulated T cell line to the peptide-loaded HLA-A∗26^+^ cell line was much lower than the response elicited by mere addition of the peptide to the T cell line without an additional antigen-presenting cell line, which indicates that CoV-SK15 may contain further T cell epitopes restricted to other HLA-I alleles in addition to CoV-SF10. Further experiments using truncated peptides derived from SK15 showed that peptide CoV-LM9 (LYYQNNVFM) was also recognized by the T cell line from individual #178 (Figure 2I) and within CoV-LM9 the minimal epitope CoV-YM8 (YYQNNVFM) could be delineated (Figure 2J). HLA-restriction analysis revealed HLA-C∗04-mediated presentation of the peptide CoV-YM8 (Figure 2K). CoV-YM8 is completely conserved between SARS-CoV-2, OC43, HKU1, and NL63, and the sequence also shows a high homology of 88% to 229E. Furthermore, CoV-YM8 is conserved in all SARS-CoV-2 variants from Alpha to the current Omicron variants (BA.2.86, JN.1, KP.2, and KP.3).

**Figure 3:**
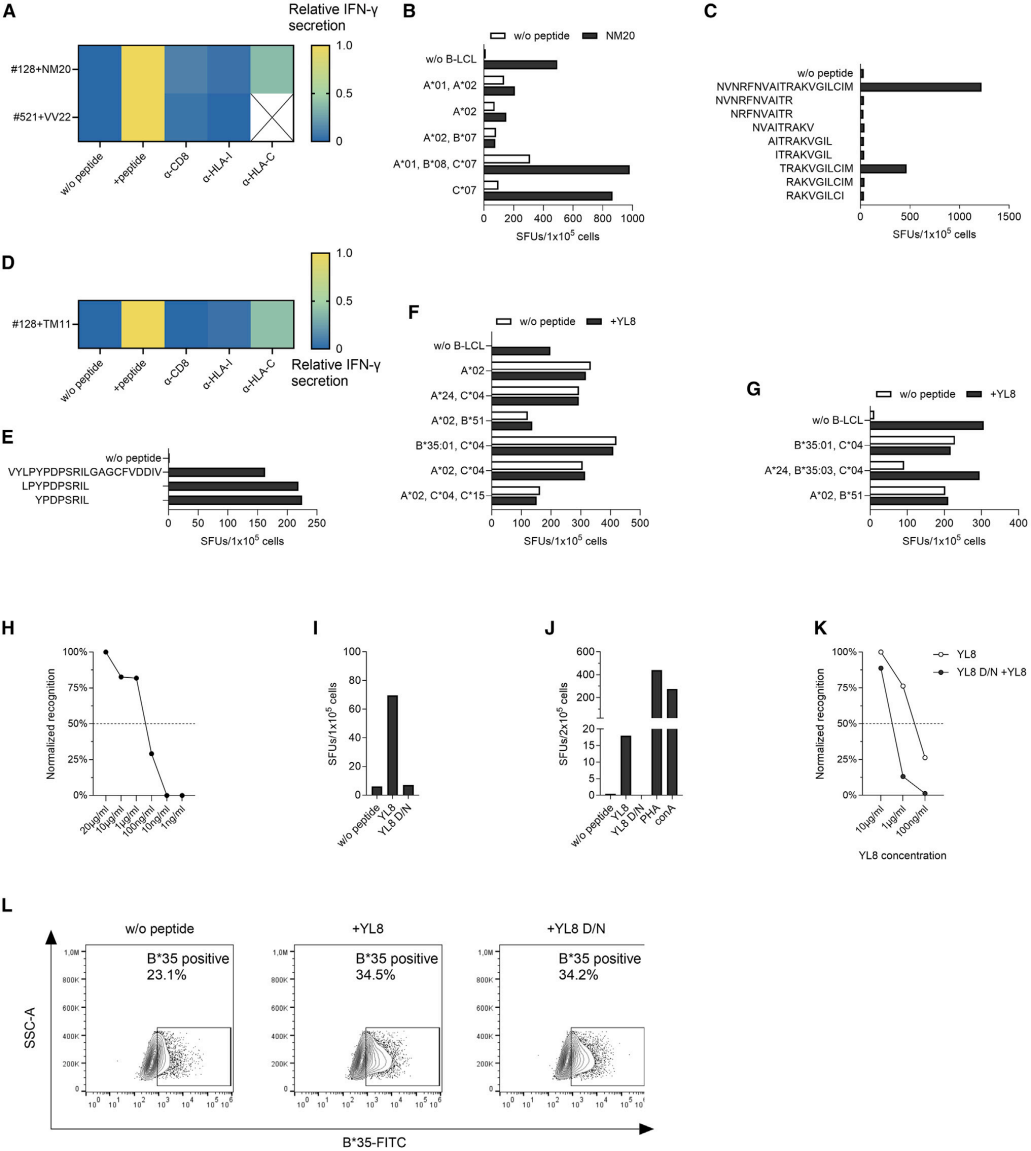
Recognition of replicase peptides by SARS-CoV-2 uninfected individuals (A) Blocking experiments with antibodies against CD8, HLA-I, and HLA-C in an IFN-γ ELISpot assay after stimulation of the T cell lines with their respective peptides. Shown are means of duplicates. (B) HLA-restriction analysis of the peptide CoV-NM20. The CoV-NM20 specific T cell line of individual #128 was co-incubated with CoV-NM20 loaded B-LCLs in an IFN-γ ELISpot. Co-incubation of B-LCLs without peptide as well as direct incubation of T cells with the peptide without B-LCLs (w/o B-LCLs) served as a control. Shown are means of duplicates. (C) Recognition of shorter peptides within CoV-NM20 sequence. CoV-NM20: NVNRFNVAITRAKVGILCIM, CoV-NR11: NVNRFNVAITR, CoV-NR9: NRFNVAITR, CoV-NV9: NVAITRAKV, CoV-AL10: AITRAKVGIL, CoV-IL9: ITRAKVGIL, CoV-TM11: TRAKVGILCIM, CoV-RM10: RAKVGILCIM, CoV-RI9: RAKVGILCI. Shown are means of duplicates. (D) Blocking experiments with antibodies against CD8, HLA-I and HLA-C in an IFN-γ ELISpot assay after stimulation of the T cell line from individual #128 with peptide CoV-TM11. Shown are means of duplicates. (E) Recognition of shorter peptides within the CoV-VV22 sequence by the T cell line from individual #521. (F and G) CoV-VV22: VYLPYPDPSRILGAGCFVDDIV, CoV-LPL10: LPYPDPSRIL, CoV-YL8: YPDPSRIL (F and G) HLA-restriction analysis of the peptide CoV-YL8. The CoV-YL8 specific T cell line YL8-1 from individual #521 was co-incubated with CoV-YL8 loaded B-LCLs in an IFN-γ ELISpot. Co-incubation of B-LCLs without peptide as well as direct incubation of T cells with the peptide without B-LCLs (w/o B-LCLs) served as a control. Shown are means of duplicates. (H) Titration curve of the peptide CoV-YL8 with the CoV-YL8 specific T cell line YL8-1 from individual #521. Shown are means of duplicates. (I and J) Recognition of the peptides CoV-YL8 and lack of recognition of CoV-YL8 D/N by (I) the CoV-YL8-specific T cell lines YL8-1 and (J) PBMCs from individual #521. Shown are means of duplicates. (K) Binding of CoV-YL8 to CoV-YL8 D/N pre-incubated cells from T cell line YL8-1 from individual #521. Measured via IFN-γ ELISpot, the SFUs were normalized to 10 μg/ml after subtraction of background. Shown are means of duplicates. (L) HLA-binding assay with CoV-YL8 and CoV-YL8 D/N on the HLA-B∗35:03^+^ Jurkat cell line. Unstimulated cells as well as unstained cells served as a control. SFUs: spot forming units. SSC-A: sideward scatter area. The prefix CoV- is omitted from the peptide names in the figure panels due to space restrictions.

Next, we performed epitope mapping in two individuals without prior SARS-CoV-2 infection who showed strong IFN-γ responses to single peptides. The T cell line from individual #128 responded to CoV-NM20 and individual #521 reacted to CoV-VV22. The IFN-γ responses toward both peptides were markedly reduced upon blockade with anti-CD8 and anti-HLA-I antibodies, suggesting the presence of HLA-I restricted epitopes within those longer peptides (Figure 3A). The CoV-NM20 directed T cell response of donor #128 could furthermore be abrogated upon HLA-C blockade, indicating an HLA-C restriction of the putative epitope.

To define the restricting HLA-I allele, we therefore co-incubated the T cell line from individual #128 with CoV-NM20-loaded B-LCLs sharing distinct HLA-I alleles. It was revealed that the T cell line recognized an HLA-C∗07 restricted epitope within CoV-NM20 (Figure 3B). Further experiments with truncated peptides showed that the peptide CoV-TM11 was recognized by the T cell line from donor #128 (Figure 3C) and the recognition was markedly reduced upon CD8, HLA-I and HLA-C blockade (Figure 3D). Abrogation of T cell recognition upon omission of the threonine at the N-terminal end and of the cysteine at the C-terminal part of the epitope demonstrated that the threonine is the N-terminal amino acid at the P1 position of the minimal epitope while the C-terminal anchor amino acid has yet to be defined. Both the cysteine, isoleucine and methionine within CoV-TM11 could serve as C-terminal anchor amino acid according to the HLA-C∗07 binding motif, which favors an arginine at the P2 position and a hydrophobic or aromatic amino acid at the C-terminal anchor position.^27^ The sequence homology of CoV-TM11 to the two beta-coronaviruses OC43 and HKU1 is 82% and 73%, respectively, while for the alpha-coronaviruses the sequence is conserved to 82% in 229E and to 73% in NL63. Like the other epitopes described in this paper, CoV-TM11 is conserved in all SARS-CoV-2 variants from Alpha to the current Omicron variants (BA.2.86, JN.1, KP.2, and KP.3).

Upon demonstrating a recognition of CoV-VV22 by CD8^+^ T cells, we performed a bioinformatic epitope prediction for potential HLA-I restricted epitopes within this peptide. High scores were yielded for the peptides CoV-LL10 (LPYPDPSRIL) and CoV-YL8 (YPDPSRIL) and a subsequent experiment allowed identification of the 8-mer CoV-YL8 as the minimal epitope (Figure 3E). The following HLA-restriction analyses with HLA-matched B-LCLs revealed that CoV-YL8 was restricted to HLA-B∗35:03 (Figures 3F and 3G), as predicted by IEDB and Syfpeithi.^25^^,^^26^ Interestingly, a peptide-loaded B∗35:01 B-LCL was not recognized by the CoV-YL8-stimulated T cell line, demonstrating the importance of the B∗35 subtype for CoV-YL8 presentation. The half-maximal peptide concentration resulting in IFN-γ production by CoV-YL8 specific T cells ranged between 1 μg/mL and 100 ng/mL (Figure 3H). CoV-YL8 was recognized in ELISpot assays not only by the CoV-YL8 stimulated T cell lines (Figure 3I) but also by freshly isolated peripheral blood mononuclear cells (PBMCs) (Figure 3J), indicating a high frequency of CoV-YL8-specific T cells in this individual. While the CoV-YL8 epitope is identical in the common cold coronavirus HKU1, the corresponding OC43 sequence contains a D to N-substitution at the P3 position abrogating recognition of the OC43 CoV-YL8 D/N peptide (YPNPSRIL) both by the T cell line (Figure 3I) and by the freshly isolated PBMCs (Figure 3J). To investigate whether the CoV-YL8 D/N variant can also bind to HLA-B∗35:03, the CoV-YL8-specific T cell line of individual #521 was pre-incubated with CoV-YL8 D/N, which greatly diminished the IFN-γ secretion elicited by CoV-YL8 (Figure 3K) and suggests binding of CoV-YL8 D/N to B∗35:03. In order to confirm those findings, an HLA-B∗35 binding assay was performed. After incubation with either CoV-YL8 or CoV-YL8 D/N, HLA-B∗35 was upregulated on the surface of the B∗35:03^+^ Jurkat cells (Figure 3L), indicating once more that both CoV-YL8 and CoV-YL8 D/N can bind to HLA-B∗35:03. Thus, the D/N variations in the CoV-YL8 epitope do not influence the ability to bind to the antigen-presenting cell but rather result in a differential TCR engagement.

Taken together, we could define several conserved SARS-CoV-2 replicase epitopes inducing CD8 T cell responses in persons with or without prior SARS-CoV-2 infection (Table 1), including CoV-YL8 that was identical to the CCCoV HKU1 sequence and elicited strong IFN-γ responses in persons without prior SARS-CoV-2 infection, thus suggesting a high cross-reactivity potential. Indeed, the CoV-YL8 sequence is also 100% conserved in NL63 and shows 88% homology to 229E.

**Table 1:** Epitopes within replicase 1ab

Peptide		Epitope		HLA restriction
Name	Sequence	Name	Sequence	
CA20	CPAEIVDTVSALVYDNKLKA	CV9	CPAEIVDTV	B∗35
SK15	SVLYYQNNVFMSEAK	SF10	SVLYYQNNVF	A∗26
		YM8	YYQNNVFM	C∗04
NM20	NVNRFNVAITRAKVGILCIM	TM11	TRAKVGILCIM	C∗07
RF16	RWVLNNDYYRSLPGVF	/	/	B∗18
VV22	VYLPYPDPSRILGAGCFVDDIV	YL8	YPDPSRIL	B∗35:03

### A YL8-specific TCR derived from a SARS-CoV-2 negative individual recognizes the YL-8 peptide after CRISPR-Cas9-mediated expression in PBMCs

**Figure 4:**
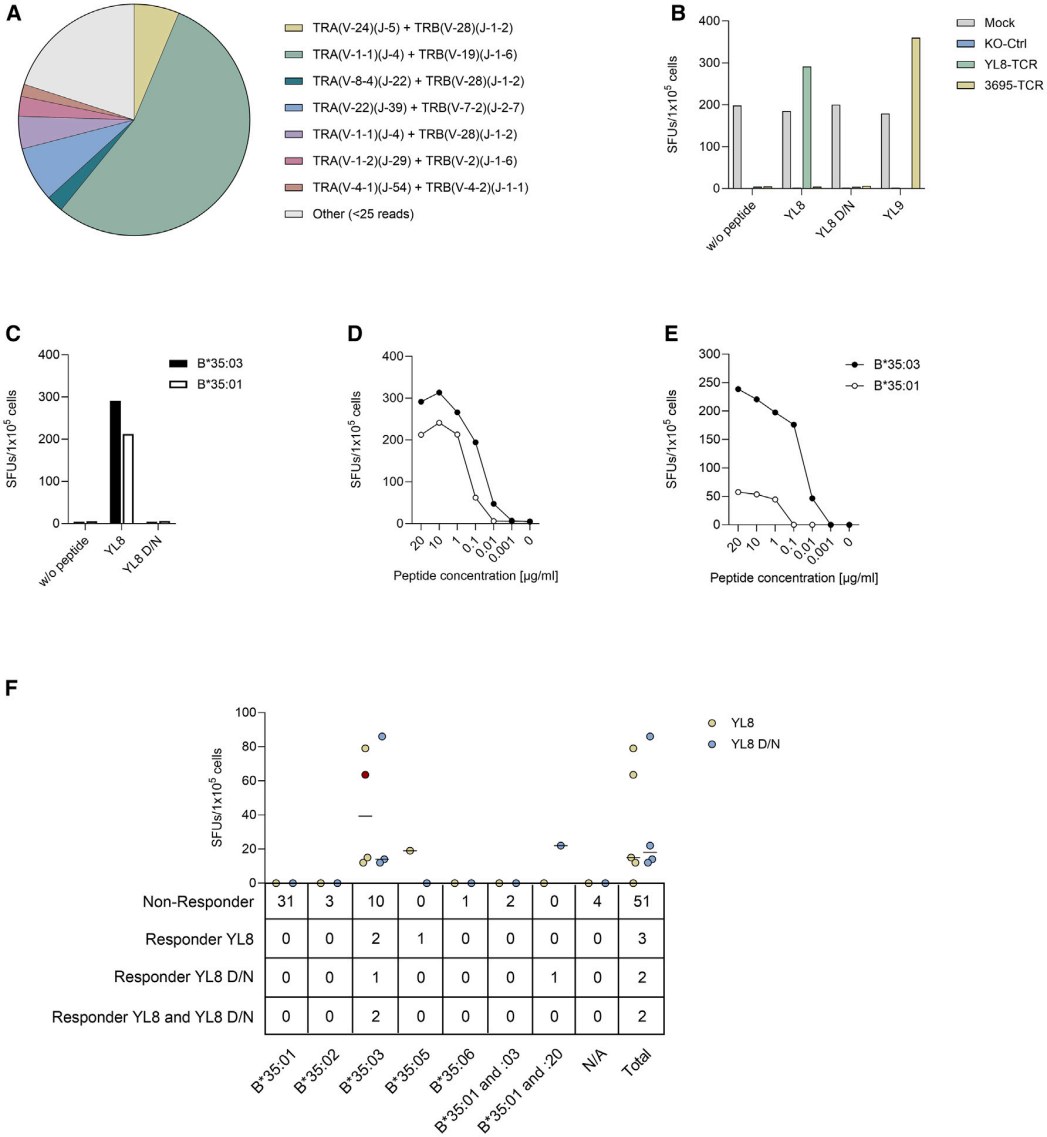
Functional analysis of CoV-YL8 TCR and CoV-YL8 responses in IFN-γ ELISpot assays in dependence on HLA-B∗35 subtype (A) TCR repertoire of the CoV-YL8 stimulated T cell line of individual #521. (B) Recognition of the peptides CoV-YL8, CoV-YL8 D/N and CoV-YL9 presented on an A∗02 B∗35:03 B-LCL by unedited (mock) cells, TCR knock-out cells (KO-Ctrl), YL8-TCR, and 3695-TCR expressing cells. Shown are means of duplicates. (C) Recognition of peptides CoV-YL8 and CoV-YL8 D/N by CoV-YL8-TCR carrying cells after presentation on a B∗35:01 or B∗35:03^+^ B-LCL. Shown are means of duplicates. (D) Titration curve of the peptide CoV-YL8 presented to CoV-YL8 TCR carrying cells by B∗35:01 or B∗35:03^+^ B-LCLs. Shown are means of duplicates and SFUs after subtraction of background (w/o peptide B∗35:03 B-LCL: 4.5 SFUs, w/o peptide B∗35:01 B-LCL: 5.5 SFUs). (E) Titration curve of the peptide CoV-YL8 presented to the YL8-specific T cell line YL8-2 from individual #521 by B∗35:01 or B∗35:03^+^ B-LCLs. Shown are means of duplicates and SFUs after subtraction of background (w/o peptide B∗35:03 B-LCL: 198 SFUs, w/o peptide B∗35:01 B-LCL: 277 SFUs). (F) Recognition of the peptides CoV-YL8 and CoV-YL8 D/N by stimulated T cells of individuals with various B∗35 subtypes. The numbers of non-responders and of responders to the indicated peptide are shown in the table. The magnitude of the recognition of the indicated peptides is depicted in the graph. Shown are SFUs after subtraction of background. Individual #521 is color-coded in red. SFUs: spot forming units. N/A: not available.

Next, we further explored the sequence and functional characteristics of a YL8-specific TCR. To this end, we made use of T cell lines from individual #521 who - up to the last evaluation time point in May 2024 and therefore three years after the first screening - had no history of COVID-19, had remained seronegative for antibodies against SARS-CoV-2 nucleocapsid and yet displayed strong CoV-YL8 responses by both fresh PBMCs and T cell lines. CD8^+^ IFN-γ^+^ T cells from the CoV-YL8 specific T cell line derived from individual #521 were sorted for single-cell TCR sequencing (Figure S2). The sorting was performed 24 months after the first detection of the response. A dominant TCR (TRA(V-1-1)(J-4) + TRB(V-19)(J-1-6)) with the CDR3 sequences CAVRGISGGYNKLIF (α chain) and CASSIEGSYNSPLHF (β chain) was identified (Figure 4A) and subsequently expressed in PBMCs of a healthy HLA-B35 negative individual with prior SARS-CoV-2 infection via CRISPR/Cas9, leading to TCR surface expression in 6.9% of the T cells (Figure S3). In contrast to the YL-8 TCR, neither the unedited nor the TCR knock-out control showed any recognition of the peptides CoV-YL8 or CoV-YL8 D/N (Figure 4B). A TCR restricted to the HLA-A∗02 spike peptide CoV-YL9 (3695-TCR^28^) served as a control and strongly recognized its cognate peptide but showed no response to CoV-YL8 or CoV-YL8 D/N, confirming the specificity of the assay (Figure 4B).

Co-incubation of the YL8 TCR-expressing PBMCs with peptide loaded B∗35:03^+^ B-LCLs showed recognition of CoV-YL8 but not CoV-YL8 D/N (Figure 4C), in line with previous responses of CoV-YL8 specific T cell lines from individual #521 (Figure 3I). Surprisingly, the CoV-YL8 TCR also recognized the peptide CoV-YL8 when presented on a B∗35:01^+^ B-LCL, albeit to a lower extent (Figures 4C and 4D). This could be due to a higher frequency of CoV-YL8-specific T cells in the TCR-edited PBMCs or due to a higher functional activity of the edited T cells that could be activated by lower amounts of peptide-HLA molecule complexes. Since we observed recognition of CoV-YL8 on a B∗35:01^+^ B-LCL by the CoV-YL8 TCR, we performed further experiments with another CoV-YL8 stimulated T cell line from individual #521. As expected, CoV-YL8 was strongly recognized when presented by a B∗35:03^+^ B-LCL while presentation of CoV-YL8 on B∗35:01 elicited a detectable, but much weaker IFN-γ response of the CoV-YL8 specific T cell line (Figure 4E). This demonstrated that the CoV-YL8 peptide could bind to HLA-B∗35:01 but presumably with a lower affinity in comparison to HLA-B∗35:03. This was also suggested by the epitope-MHC affinity prediction of the epitope CoV-YL8 by the database IEDB, yielding a higher score for B∗35:03 (0.836) compared to B∗35:01 (0.507).^26^

To assess the frequency of recognition of the CoV-YL8 epitope and to investigate whether TCRs from other individuals may recognize CoV-YL8 D/N, we stimulated T cell lines from 58 HLA-B∗35:03^+^ individuals (including individual #521) with the peptides CoV-YL8 and CoV-YL8 D/N and tested outgrowing T cells for peptide recognition. Three individuals were PLWOH, the other 55 were PLWH. Out of the 15 B∗35:03^+^ individuals, two responded to CoV-YL8 only, one to CoV-YL8 D/N only and two to both peptides (Figure 4F). One individual with HLA-B∗35:05 responded to CoV-YL8. One PLWOH possessing both the B∗35:01 and B∗35:20 alleles recognized CoV-YL8 D/N but not CoV-YL8. We assume presentation of the CoV-YL8 D/N peptide by the B∗35:20 allele but due to the low frequency of B∗35:20 this could not be proven experimentally. Importantly, none of the individuals carrying only the B∗35:01 allele responded to either of the peptides. The recognition of CoV-YL8 D/N by some individuals indicates that the peptide can be presented by HLA-B∗35:03 and that lack of recognition of YL8 D/N by individual #521 was caused by insufficient interaction between the TCR and the peptide-HLA-complex.

### Distinct HLA-I alleles are associated with increased or decreased SARS-CoV-2 susceptibility

The CoV-YL8 epitope is completely conserved in the main SARS-CoV-2 variants from Alpha up to the latest Omicron variants (BA.2.86, JN.1, KP.2, and KP.3) with the exception of the Delta 21J variant that contained an L to I substitution at the P9-position of the epitope in 8.43% of the sequences (https://covariants.org/variants/21J.Delta).^29^

**Figure 5:**
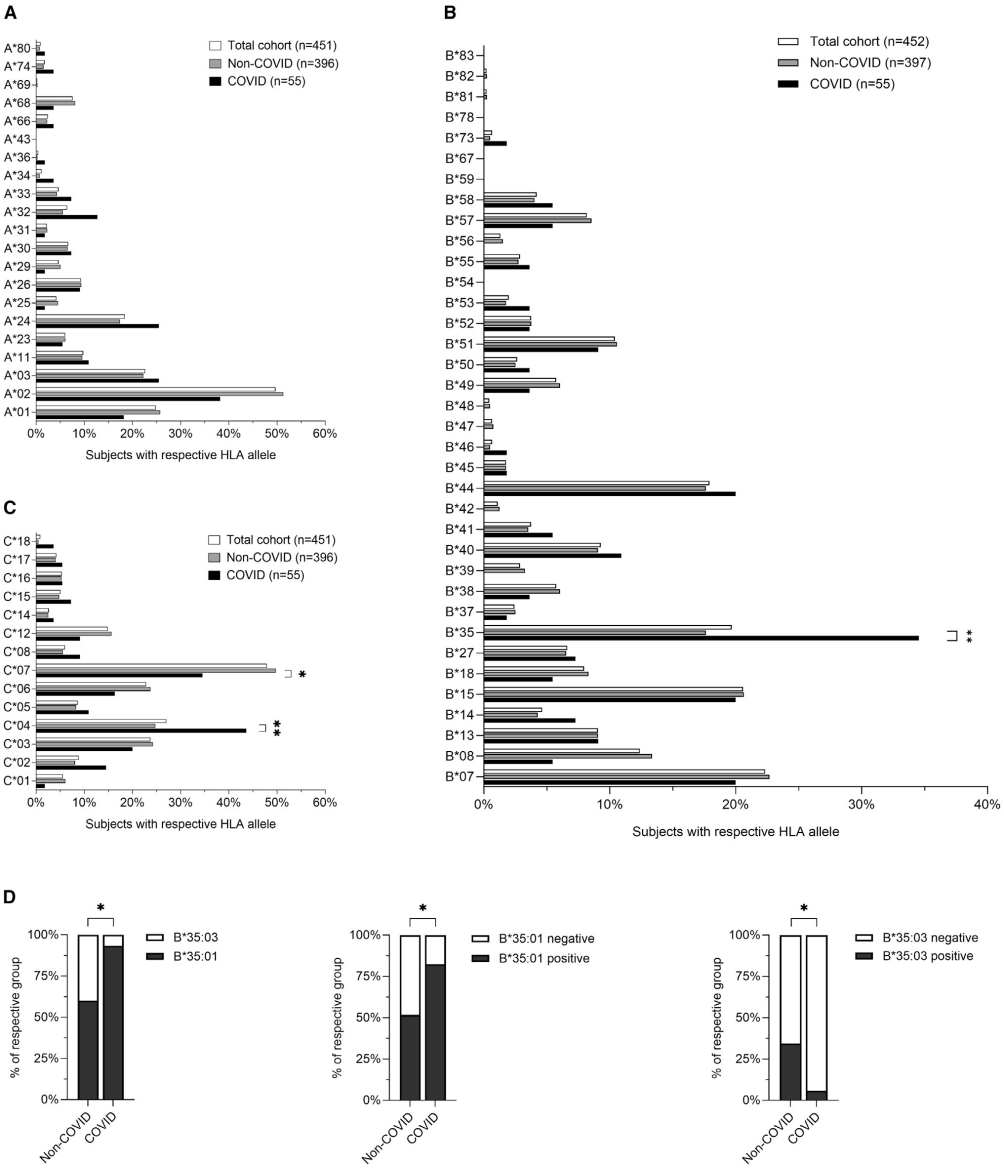
Association of HLA-I alleles with SARS-CoV-2 infection (A–C) Association of (A) HLA-A alleles, (B) HLA-B alleles, and (C) HLA-C alleles with SARS-CoV-2 infection. (D) Frequencies of HLA-B∗35 subtypes according to SARS-CoV-2 infection status within the HLA-B∗35^+^ individuals of the Erlangen HIV-1 cohort. *n* B∗35 = 75, *n* B∗35:01 = 44, *n* B∗35:03 = 21, *n* other B∗35 subtypes = 10. For statistical comparisons, two-tailed Fisher’s exact tests were used. Non-COVID: individuals without SARS-CoV-2 infections, COVID: individuals with at least one SARS-CoV-2 infection. One individual was only typed for the HLA-B locus. The n for each HLA-I allele is shown in Table S3. ∗ = p ≤ 0.05, ∗∗ = p ≤ 0.01.

Our observation that several B∗35:03 individuals without prior SARS-CoV-2 infection had T cell IFN-γ responses against the epitope CoV-YL8 prompted us to explore the potential effect of HLA-I alleles on the susceptibility to SARS-CoV-2 in the Erlangen HIV-1 cohort (Figures 5A–5C).

SARS-CoV-2 infection rates assessed during regular clinical visits detected SARS-CoV-2 infections in 55 out of 452 PLWH between March 1^st^ 2020 and June 30^th^ 2021 (Figure S4). The SARS-CoV-2 infected group (COVID group) displayed a significant overrepresentation of both HLA-B∗35 (Odds ratio: 2.465; Figure 5B) and the linked HLA-C∗04 (Odds ratio: 2.354, Figure 5 C), while HLA-C∗07 was significantly underrepresented in the COVID group (Odds ratio: 0.533, Figure 5C). Importantly, age, viral load, CD8 counts as well as the CD4/CD8 ratio did not differ between HLA-B∗35, HLA-C∗04, or HLA-C∗07 -positive and negative individuals (Figure S5). The CD4 T cell count also did not differ between HLA-C∗04 or HLA-C∗07 -positive and negative individuals but was higher in the HLA-B∗35^+^ individuals (median 720/μL for B∗35^+^, median 645/μL for B∗35^-^, *p* = 0.0497, Figure S5).

As we had observed differences between HLA-B∗35:01 and HLA-B∗35:03 regarding the recognition of the CoV-YL8 peptide, we performed a more detailed analysis of the B∗35 subtypes with exclusion of all individuals that were not subtyped for HLA-B∗35 or heterozygous for the B∗35 locus. This revealed that HLA-B∗35:01 was significantly overrepresented in the SARS-CoV-2 infected group (Figure 5D). On the other hand, HLA-B∗35:03 was significantly overrepresented in the non-COVID group, suggesting a protective effect of this HLA allele (Figure 5D). Importantly, B∗35:01 and B∗35:03 individuals did not differ significantly in CD4 counts, CD8 counts, CD4/8 ratio, viral load, age, or numbers of SARS-CoV-2 vaccinations (Figure S6).

## Discussion

Our study shows that SARS-CoV-2 replicase specific T cells are present in a considerable number of ART-treated PLWH without prior SARS-CoV-2 infection, presumably due to the high sequence homology between SARS-CoV-2 and CCCoVs. Although limited, a few studies suggest the importance of cross-reactive T cells for SARS-CoV-2 susceptibility. Recognition of a cross-reactive HLA-B∗07-restricted nucleocapsid epitope was associated with a milder course of COVID-19^9^^,^^30^ and significantly higher frequencies of cross-reactive nucleocapsid-specific memory T cells were reported in people who stayed PCR-negative after SARS-CoV-2 exposure.^11^ An expansion of pre-existing SARS-CoV-2 specific T cells was detected in medical personnel with abortive seronegative SARS-CoV-2 infections.^10^ In that study, T cells from pre-pandemic time points and from seronegative health care workers preferentially targeted the RNA polymerase. In a few of these prior studies, cross-reactive T cells against epitopes in the replicase were described in PLWOH persons^10^^,^^11^^,^^12^^,^^17^^,^^31^^,^^32^ and some of those epitopes are neighboring, but different from the epitopes identified in our study on people with HIV-1. Thus, while cross-reactivity may target the same broad region, our findings underscore the possibility of distinct pan-coronaviruses cross-reactive T cell epitopes between PLWH and PLWOH.

In this study, we focused on PLWH. As most of the patients of the Erlangen HIV cohort were HLA-I typed and came to their regular visits even during the lock-downs, we had the unique chance to perform a prospective study from the beginning of the SARS-CoV-2 epidemic on with a large cohort of HLA-typed individuals. However, due to the limitations of the early COVID-19 epidemic, it was not possible to establish a similar cohort of HLA-I typed PLWOH that could be investigated at regular visits. Furthermore, while a meta-analysis of the interaction between HIV-1 and SARS-CoV-2 infection indicated that PLWH exhibited no increased risk of contracting SARS-CoV-2 infection, they did have a higher risk of developing severe COVID-19 and a higher mortality than PLWOH.^33^ Untreated HIV-1 infection has a strong negative effect on SARS-CoV-2 immunity that can be at least partially compensated by antiretroviral therapy. In the study of Karim et al., untreated PLWH with HIV-1-viremia and low CD4 counts developed a more prolonged course of SARS-CoV-2-infection in contrast to ART-treated PLWH or PLWOH.^34^ In that report, SARS-CoV-2-spike specific CD4^+^ T cells and CD8^+^ T cells were mostly undetectable in untreated PLWH with HIV-1 viremia and low CD4 counts but they were frequently detected in PLWH on ART. SARS-CoV-2-specific T cell responses after mild or moderate COVID-19 were observed at similar frequencies in the majority of PLWH on ART and healthy controls by Alrubayyi et al*.*^35^ In that study, SARS-CoV-2-specific CD4^+^ T cells exceeded SARS-CoV-2-specific CD8^+^ T cells and SARS-CoV-2-specific T cells showed stronger responses to spike, membrane and nucleoprotein than to regulatory proteins, however, T cells against replicase were not analyzed. A lower interleukin 2 (IL-2) production of SARS-CoV-2-specific CD8^+^ T cells targeting non-spike functional antigens indicated that there still are functional alterations in the CD8 cell compartment in ART-treated PLWH.

Although Helftdal et al. reported lower BNT162b2-induced T cell responses in PLWH only at low CD4 T cell counts,^36^ numerous studies observed lower humoral and cellular immune responses toward the SARS-CoV-2 spike protein in vaccinated PLWH compared to PLWOH.^37^^,^^38^^,^^39^^,^^40^ Therefore, incorporation of epitopes that are particularly well recognized in people living with HIV may help close the gap in immunogenicity observed thus far.

Aside from identifying cross-reactive epitopes, TCR repertoire analysis has emerged as powerful tool in vaccine research as it allows to determine which TCR clonotypes are associated with a high response rate. Similar to what has been observed for other pathogens, TCR repertoire analysis in SARS-CoV-2 other studies reported that the majority of identified SARS-CoV-2 specific or cross-reactive TCRs were private, rather than public clonotypes^41^^,^^42^ with some TCR clones associating with protection against COVID-19.^41^ In our study, we identified the dominant YL8-specific TCR in a donor that to date has never contracted SARS-CoV-2. An *in-silico* search in TCR-databases did not yield any hits for this YL8-specific TCR, indicating it is likely a private rather than a public TCR. While public TCRs are rare, some shared SARS-CoV-2 specific or cross-reactive TCR clonotypes associating with CD4 T cell epitopes have been reported.^42^

Next to the epitope repertoire and corresponding TCRs, HLA alleles are known to determine T cell responses to SARS-CoV-2. The strength of the peptide-HLA interaction is a key factor in mediating the T cell response toward epitopes, as a higher HLA-I-peptide stability is associated with increased antigen immunogenicity.^43^^,^^44^ In the case of HIV-1, it has been shown that immunodominant epitopes lead to a higher stability of the peptide-HLA complex and that protective HLA-I alleles are to a greater extent stabilized by highly networked epitopes compared to non-protective alleles.^45^ For SARS-CoV-2, *in-silico* predictions revealed a high variance in the number of peptides binding to single HLA-I alleles,^46^ which may at least in part explain the observation that certain HLA-I alleles confer increased or decreased susceptibility to SARS-CoV-2 infection or the course of COVID-19.^47^^,^^48^^,^^49^^,^^50^^,^^51^^,^^52^^,^^53^ In line with our observations, HLA-C∗07 has been linked to a milder course of disease in other studies^47^^,^^48^ and several studies also showed that HLA-B∗35 and HLA-C∗04—which is in linkage disequilibrium with HLA-B∗35—led to an increased risk of SARS-CoV-2 infection or a more severe disease course.^47^^,^^48^^,^^49^^,^^50^^,^^51^ However, Naemi et al. and Fischer et al. described a protective effect of the alleles HLA-B∗35 and HLA-B∗35:01.^52^^,^^53^ Instead, several other HLA-I alleles such as HLA-A∗02, B∗15, B∗51, or C∗14 were linked to SARS-CoV-2 susceptibility or disease severity.^52^^,^^53^ The reasons for the differences regarding the associations of certain HLA-alleles to disease susceptibility or disease severity are not yet clear. However, various factors such as ethnicity, cohort size, infection rates and emergence of SARS-CoV-2 variants, vaccination status, and adaption of viral sequences over time to certain HLA alleles may play a role. As our HIV-1 cohort could have been enriched for some HLA-I alleles due to the fact that certain HLA-I alleles either accelerate or slow down HIV-1 disease progression,^54^ the lack of a link between SARS-CoV-2 susceptibility and HLA-I alleles that were rare in our study would need to be confirmed in larger HIV-1 populations where those alleles are sufficiently represented.

An important finding of our study was the contrasting effects of the HLA-B∗35 subtypes HLA-B∗35:01 and B∗35:03. Both HLA-B∗35:01 and B∗35:03 alleles differ by only one amino acid at position 116 which is located at the base of the F pocket in the peptide binding cleft of the HLA-B∗35 molecule. In the HLA-B∗35:01 molecule, a serine at position 116 favors the binding of large aromatic residues such as tyrosine or phenylalanine, whereas in the HLA-B∗35:03 molecule, a phenylalanine at position 116 limits the binding of large aromatic amino acids and facilitates binding of small hydrophobic amino acids such as leucine.^55^ Thus, within the same allele group, minor substitutions could alter the effect of the HLA type on T cell responses. This underlines the necessity to perform high-resolution HLA typing to study the role of HLA genes in infectious diseases.

In conclusion, our study demonstrates frequent T cell recognition of SARS-CoV-2 replicase in treated PLWH without prior SARS-CoV-2 infection as well as a relationship between HLA-I types and COVID-19 susceptibility in people with HIV-1. The recognition of cross-reactive epitopes, such as the newly identified HLA-B∗35:03-restricted T cell epitope CoV-YL8, could contribute to our observation that HLA-B∗35:03 associated with a lower rate of SARS-CoV-2 infection in our cohort. Our findings of frequent cross-reactivity between SARS-CoV-2 and homologous beta-coronaviruses, such as OC43 and HKU1^56^ opens perspective for a SARS-CoV-2 vaccine that may induce strong T cell responses in people with HIV-1 not only against SARS-CoV-2 but also to other betacoronaviruses. While expansion of pre-existing SARS-CoV-2-specific T cells targeting epitopes in the replicase has been associated with abortive seronegative SARS-CoV-2-infections,^10^ further studies are needed to explore whether immunization against cross-reactive epitopes could influence the rate and the course of infection by other betacoronaviruses in individuals displaying the corresponding epitope-presenting HLA-alleles.

### Limitations of the study

A limitation of our study is that we only focused on CD8 T cell epitopes and HLA-I alleles, therefore we could not report on the potential CCCoV/SARS-CoV-2 cross-reactive CD4 T cell responses in people with HIV-1. Furthermore, a potential effect of HLA-II alleles on SARS-CoV-2 susceptibility could not be assessed and the effect of rare HLA-I alleles would need to be studied in a larger cohort. A further limitation is the lack of an HIV-1 negative control group and therefore our findings cannot be directly transferred to the general population.

## Resource availability

### Lead contact

Further information and requests for resources and reagents should be directed to and will be fulfilled by the lead contact Prof. Thomas Harrer (Thomas.harrer@uk-erlangen.de).

### Materials availability

Resources and reagents generated in this study will be made available by the lead contact upon request following publication by reasonable formal request from qualified researchers, subject to a signed data sharing agreement and in compliance with the requirements of the funding bodies and institutions.

### Data and code availability

• Anonymised data are available from the lead contact upon request following publication by reasonable formal request from qualified researchers, subject to a signed data sharing agreement and in compliance with the requirements of the funding bodies and institutions.
• This paper does not report original code.
• Any additional information required to reanalyze the data reported in this article is available from the lead contact upon request.

## Acknowledgments

We thank Uwe Appelt for sorting the T-cell lines and Katharina Kocher for providing the SARS-CoV-2 #3695 TCR construct. Data of this manuscript were part of the PhD thesis of KGS and the MD thesis of P.G. This work was funded by the H.W. & J. Hector Foundation, project M2102 (T.H., K.N.-M.), the Interdisciplinary Centre for Clinical Research (IZKF) Erlangen, project TP A94 (A.E.), the coronavirus research grant by the Bavarian State Ministry of Health Bay-VOC (A.E.) and the German Federal Ministry of Education and Research (BMBF), project 01KI2013 (K.S. and E.-M.S.).

## Author contributions

Conceptualization, K.G.S., K.N.-M., and T.H.; methodology, K.G.S., E.-M.S., K.S., K.N.-M., and T.H.; investigation, K.G.S., P.G., E.-M.S., C.S., E.G.H., M.L., B.S., P.S., S.B., and A.E.; resources, E.G.H., V.S., and T.H.; data curation, K.G.S., A.E., and K.S.; formal analysis, K.G.S. and A.E.; validation, K.G.S., K.N.-M., and T.H.; visualization, K.G.S., K.N.-M., and T.H.; funding acquisition, A.E., K.S., K.N.-M., and T.H.; project administration, K.N.-M. and T.H.; supervision, K.N.-M., and T.H.; writing – original draft, K.G.S, K.N.-M., and T.H.; writing – review and editing, all authors.

## Declaration of interests

The authors declare no competing interests.

## STAR★Methods

### Key resources table

**Table:** 

REAGENT or RESOURCE	SOURCE	IDENTIFIER
**Antibodies**		

Purified mouse anti-human CD8 Antibody, Clone SK1	BioLegend	Cat# 344702; RRID:AB_1877104
Purified mouse anti-human HLA-A,B,C Antibody, Clone w6/32	BioLegend	Cat# 311402; RRID:AB_314871
Anti-human B17, B35, B44+ (IgM)	OneLambda	Cat#0786BHA
Anti-human IFN-γ mAb (1-D1K), unconjugated	Mabtech	Cat# 3420-3-1000; RRID:AB_907282
Anti-human IFN-γ mAb (7-B6-1), biotin	Mabtech	Cat# 3420-6-1000; RRID:AB_907272
Goat Anti-Mouse IgM-FITC	SouthernBiotech	Cat#1021-02; RRID:AB_2794237
Brilliant Violet 421™ anti-human CD8 Antibody, Clone SK1	BioLegend	Cat# 344748; RRID:AB_2629584
TotalSeq™-C0258 anti-human Hashtag 8 Antibody	BioLegend	Cat# 394675; RRID:AB_2820044
APC/Fire™ 750 anti-mouse TCR β chain Antibody, Clone H57-597	BioLegend	Cat#109246; RRID:AB_2629697
Purified anti-human CD3 (clone OKT3)	BioLegend	Cat#317302; RRID:AB_571927
Purified anti-human CD28 (clone CD28.2)	BioLegend	Cat#302902; RRID:AB_314304
FITC anti-human TCR α/β (Clone IP26, 100T)	BioLegend	Cat#306706; RRID:AB_314644
Anti-human CD8a-eF450 (clone OKT8)	Life Technologies	Cat#48-0086-42; RRID:AB_1907412
Anti-human CD4-PE (clone RPA-T4) monoclonal antibody	Life Technologies	Cat#12-0049-42; RRID:AB_1582249

**Biological samples**		

Peripheral blood mononuclear cells	Healthy volunteers and HIV positive donors, UK Erlangen	

**Chemicals, peptides, and recombinant proteins**		

RPMI-1640	ThermoFisher	Cat#21875091
PBS	ThermoFisher	Cat#10010023
FCS, Lot AN-S00FG	Anprotec	Cat#AC-SM-0027
FBS	ThermoFisher	Cat#10270106
Penicillin/Streptomycin 10000 U/mL	ThermoFisher	Cat#15140122
L-Glutamin (GlutaMAX)	ThermoFisher	Cat#35050061
HEPES	Merck Millipore	Cat#83264-500ML-F
Human AB serum	Merck Millipore	Cat#H4522-100ML
Gentamicin 50 mg/mL	Life Technologies	Cat#15750060
2-Mercaptoethanol 50mM	Life Technologies	Cat#31350010
HEPES	Carl Roth	Cat#HN77.3
L-Glutamine	Sigma-Aldrich	Cat#G8540-25g
Recombinant human IL-7, 50ug	peproTech	Cat#200-07-50
Recombinant human IL-15, 50ug	peproTech	Cat#200-15-50
Recombinant Human IL-2; 250μg	peproTech	Cat#200-02-250
Murine cGAS inhibitor RU.521	Invivogen	Cat#inh-ru521
Trichostatin A (TSA)	Abmole	Cat#M1753
M-3814 Nedisertib	Chemietek	Cat#CT-M3814
Poly-L-Glutamic Acid Sodium (PGA)	Sigma-Aldrich	Cat#P4761-100mg
Alt-R S.p. Cas9 Nuclease V3	IDT	1081059
Alt-R Cas9 Electroporation Enhancer	IDT	Cat#10007805
Zombie Aqua Fixable Viability Kit	BioLegend	Cat#423101
BSA, Fraktion V; biotinfrei	Carl Roth	Cat#163.4
Water, nuclease free	Life Technologies	Cat#R0582
Herculase II Fusion DNA Polymerase	Agilent	Cat#600677
dNTP Mix (10mM each) 1mL	Life technologies	Cat#R0192
3-Amino-9-Ethyl-Carbazole (AEC)	Sigma-Aldrich	Cat#A5754-100G
Biocoll	Bio&Sell	Cat#BS.L6115
Cyclosporin	Sigma-Aldrich	Cat#30024-25MG
Dimethyl sulfoxide (DMSO)	Carl Roth	Cat#A994.2
Dithiothreitol (DTT)	Sigma-Aldrich	Cat#1114740001
Ethylendiamintetraacetate (EDTA)	Merck Millipore	Cat#8043.2
Methanol	Carl Roth	Cat#T909.1
N,N-Dimethylformamide (DMF)	Merck Millipore	Cat#D4551-500ML
Paraformaldehyde (PFA)	Carl Roth	Cat#0964.1
Saponin	Merck Millipore	Cat#47036-50G-F
Tween 20	Merck Millipore	Cat#P1379-500ML
H_2_O_2_	Merck Millipore	Cat#31642-500ML-M
Peptides	Intavis Peptide Services	
Peptides	EMC Microcollections	
PHA	Merck Millipore	Cat#L4144-5MG

**Critical commercial assays**		

IFN-γ Secretion Assay – Detection Kit (PE), human, for 100 tests	Miltenyi	Cat#130-054-202
SARS-CoV-2 Spike IgG ELISA	Euroimmun	Cat#EI 2606–9601 G
SARS-CoV-2 NCP IgG ELISA	Euroimmun	Cat#EI 2606-9601-2 G
VECTASTAIN Elite ABC-HRP Kit	Vector Laboratories	Cat# PK-6100
Chromium Next GEM Single-Cell 5′ kit v.2	10x Genomics	Cat#PN-1000263
MinElute PCR Purification Kit (50)	Qiagen	Cat#28004
P3 Primary Cell 96-well Nucleofector Kit	Lonza	Cat#V4SP-3960
LIAISON® SARS-CoV-2 TrimericS IgG Assay	DiaSorin	Cat#311510

**Experimental models: Cell lines**		

Jurkat cells	J. Dörrie, Department of Dermatology, University hospital Erlangen	RRID:CVCL_0065
B95-8	DSMZ	RRID:CVCL_1953

**Oligonucleotides**		

crRNA TRAC6 (5′-AGAGTCTCTC AGCTGGTACA-3′)	IDT	
crRNA TRBC3 (5′-GGAGAATGA CGAGTGGACCC-3′)	IDT	
Alt-R® CRISPR-Cas9 tracrRNA, 100 nmol	IDT	Cat#1072534
hTRAC HDR genomic rev (CATCATTGACCAGAGCTCTG)	ThermoFisher	
hTRAC tCTS LHA fwd (TCTCTCTCTCAGCTGG TACACGGCTGCCTTTACTCTGCCAGAG)	ThermoFisher	

**Recombinant DNA**		

HDR DNA template sequence	Schober et al.^57^	

**Software and algorithms**		

AID EliSpot 8.0	AID Autoimmun Diagnostika	
FlowJo, version 10.7.2	BD Biosciences	RRID:SCR_008520; https://www.flowjo.com/solutions/flowjo/downloads
GraphPad Prism, version 9	GraphPad Software	RRID:SCR_002798; https://www.graphpad.com/features
Magellan™, version 7.1	TECAN	https://lifesciences.tecan.com/software-magellan
Soarian Clinicals	Siemens	
Navios software	Beckman-Coulter	RRID:SCR_014421
CLC Genomics Workbench 23.0.3	Qiagen	RRID:SCR_011853; https://digitalinsights.qiagen.com/products-overview/discovery-insights-portfolio/analysis-and-visualization/qiagen-clc-workbench-premium/?cmpid=QDI_GA_DISC_CLC&gad_source=1&gclid=Cj0KCQjw05i4BhDiARIsAB_2wfAfwpKDq5aCgT1VqSuz2omIorAn87lt4ar-Jd5RvyJJDdMN0wI75VsaAmlEEALw_wcB
BD FACSDiva Software	BD Biosciences	RRID:SCR_001456; https://www.bdbiosciences.com/en-us/products/software/instrument-software/bd-facsdiva-software
SBTengine	GenDx	
MiaFora 5.2	Immucor	
Uniprot	The UniProt Consortium	RRID:SCR_002380

### Experimental model and study participant details

#### Study participants and design

The objective of this study was to analyze SARS-CoV-2 replicase cross-reactive T cells in relation to SARS-CoV-2 susceptibility as well as the influence of HLA-I types in PLWH. This study took place during the SARS-CoV-2 epidemic, during which several lockdowns severely hindered the possibility of including HIV-1-uninfected persons. The continuity in the clinical care of PLWH who maintained regular visits despite lockdowns, allowed us to explore T cell cross-reactivity specifically in this demographic group, for whom little data were available. For this purpose, we recruited 177 PLWH in whom SARS-CoV-2 prior exposure was assessed by measurement of anti-spike IgG antibodies in 50 unvaccinated individuals and by measurement of anti-nucleocapsid IgG antibodies in 127 vaccinated individuals. Detailed clinical characteristics of the individuals can be found in Table S4. Inclusion criteria for the 177 individuals were HIV-1 infection, cART, no HIV-1 unrelated immunodeficiencies or overt diseases. SARS-CoV-2 spike-specific IgG were detected using the LIAISON SARS-CoV-2 TrimericS IgG assay (Diasorin, Dietzenbach, Germany) or the Euroimmun SARS-CoV-2 IgG ELISA, Lübeck, Germany).^58^^,^^59^ According to the manufacturer’s instructions, LIAISON binding antibody units (BAU)/mL Values above ≥33.8 and a Euroimmun ratio above 1.1 were considered positive. A ratio of 0.8–1.1 in the Euroimmun assay was considered borderline. SARS-CoV-2 nucleocapsid-specific IgG were detected using the Abbott ARCHITECT SARS-CoV-2 IgG CMIA^60^ with ratios above 1.4 considered positive and ratios between 0.4 and 1.39 considered borderline. Antibody measurements were performed once in unicates. Of the 177 individuals, 133 (75%) had no indications of prior SARS-CoV-2 infections as assessed by medical history, absence of SARS-CoV-2 spike or nucleocapsid antibodies in vaccinated and unvaccinated individuals, respectively. Nucleocapsid serology or SARS-CoV-2 PCR determined prior infection in 44 of the 177 individuals. To analyze the rates of SARS-CoV-2 infection in dependence of HLA-I type among people with HIV-1, incidences of SARS-CoV-2 infections were collected between 1^st^ of March 2020 and 30^th^ of June 2021 in 452 HIV-1^+^ individuals, including the 163 HLA-1 typed individuals of the 177 individuals analyzed for recognition of the replicase peptides. Past SARS-CoV-2 infections were determined upon proof of detectable SARS-CoV-2 RNA, anti-SARS-CoV-2 spike specific antibodies in the absence of vaccination or anti-SARS-CoV-2 nucleocapsid antibodies. Inclusion criteria for the 452 individuals were HIV-1 infection and a follow-up visit in the outpatient clinic of the Department of Internal Medicine 3 of the University Hospital Erlangen between 1^st^ of March – 30^th^ of June 2021. Except for four individuals, all PLWH were on antiretroviral therapy. Of the 452 individuals, 352 (77.9%) were Caucasian, 49 (10.8%) African, 22 (4.9%) Asian, ten (2.2%) Turkish, eight (1.8%) Arabic, seven (1.6%) Mediterranean, three (0.7%) Hispanic, and one (0.2%) of other ethnic origin. Detailed information about these individuals can be found in Table S5. The study with analysis of virus-specific immune responses was approved by the Ethics Committee of the Medical Faculty (Number 250_15B and 157_20B). Blood was obtained after written informed consent.

#### Primary cell cultures

Human peripheral blood mononuclear cells (PBMCs) were cultured at 37°C and 5% CO_2_ in R10 medium (RPMI 1640, 10% FCS, 1% L-glutamine, 1% HEPES, 1% Pen/Strep) unless another cell culture medium is specified. T cell lines generated from PBMCs were cultured at 37°C and 5% CO_2_ in R10IL2 medium (RPMI 1640, 10% FCS, 1% L-glutamine, 1% HEPES, 1% Pen/Strep, 1 × 10^3^ U/ml IL2). PBMCs were obtained from male and female donors.

#### Cell lines

B-Lymphoblastoid cell lines (B-LCLs) were cultured at 37°C and 5% CO_2_ in R20 medium (RPMI 1640, 20% FCS, 1% L-glutamine, 1% HEPES, 1% Pen/Strep). Cells were split 2:3 every 3–4 days. B-LCLs were generated in our lab from human PBMCs from both male and female donors. As the cell lines were established by our lab, no authentication was performed. All B-LCLs tested negative for mycoplasma contamination.

Jurkats cells were cultured at 37°C and 5% CO_2_ in R10 medium and split 1:2 every 2–3 days. The Jurkat cell line was kindly provided by J. Dörrie, Department of Dermatology, University Hospital Erlangen and is derived from a 14-year old human male. Jurkat cells were authenticated by sequencing of the TCR α and β chain, the obtained sequences were then confirmed to correspond to published sequences of the Jurkat cell line. The Jurkat cell line tested negative for mycoplasma contamination.

### Method details

#### Peptides

Peptides were synthesized as crude peptide and C-terminal acids with a purity of >70% confirmed by ESI-LCMS (EMC Microcollections, Tübingen, Germany; Intavis Peptide Services GmbH, Tübingen, Germany). Peptides were dissolved in H_2_O with 10% DMSO (Merck Millipore, Darmstadt, Germany) and 1% DTT (Sigma-Aldrich, Steinheim, Germany). Peptide names refer to the first and last amino acid and the number of amino acids. To screen for homologous peptides in the SARS-CoV-2 replicase, an alignment of the SARS-CoV-2 replicase (Uniprot: P0DTD1), the OC43 replicase (Uniprot: P0C6X6), the HKU1 replicase (Uniprot: P0C6X2), the NL63 replicase (Uniprot: P0C6X5) and the 229E replicase (Uniprot: P0C6X1) was performed using the Clustal Omega program (CLUSTAL O(1.2.4) multiple sequence alignment). The SARS-CoV-2 peptides used in this study had >73% homology (median 90%, range 73–100%) to the respective OC43 or HKU1 sequence. The peptides were assigned to the pools according to their position in the replicase sequence. The first five peptides in the sequence make up pool 1, pool 2 consists of peptides 6–10, pool 3 consists of peptides 11–15 and pool 4 of peptides 16–21.

#### Isolation of PBMCs

Isolation of PBMCs was carried out via density gradient centrifugation. Leucosep tubes (Greiner Bio One GmbH, Frickenhausen, Germany) were filled with 15mL Biocoll (Biosell) and centrifuged briefly to collect the fluid under the membrane. A maximum of 35mL citrate blood was transferred to the tube and filled up to 50mL with PBS, if necessary. Cells were centrifuged at 760 × g for 20min and the upper layer containing PBMCs was transferred to 50mL tubes and centrifuged at 610 × g for 10min. The cell pellet was then washed with 30mL PBS solution at 610 × g for 10min prior to resuspension in the appropriate cell culture medium.

#### Generation of B-LCLs

Following density gradient centrifugation, 10 × 10^6^ PBMCs were resuspended in 3-4mL of sterilely filtered supernatant from the EBV-producing B95-8 cell line. Cyclosporine was added in a final concentration of 30 ng/ml and the cells were transferred to T25 flasks. After 2–3 days, R20 medium and fresh cyclosporine were added.

#### Generation of SARS-CoV-2 specific T cell lines

For the generation of specific T cell lines, 10 × 10^6^ PBMCs per 1mL of R10IL2 were stimulated with peptides in a final concentration of 4μg/ml. After 7–12 days, outgrowing cells were tested for recognition of peptides by Interferon- γ Enzyme-Linked ImmunoSpot assay (IFN-γ ELISpot).

#### Restimulation of T cell lines with APCs

For long-term propagation, T cells had to be restimulated after 3–5 weeks by stimulation with peptide loaded HLA-matched B-LCLs. Before co-cultivation, the B-LCLs were loaded with the respective peptide for 1 h, then washed once with 15mL PBS (300xg, 10min) and then irradiated with 90 Gray. PBMCs irradiated with 30 Gray were used as feeder cells. Per 1 × 10^6^ cells of the T cell line, 1 × 10^6^ irradiated B-LCLs and 1-2 × 10^6^ irradiated PBMCs were added in R10IL2. The restimulated T cell lines were incubated for at least 7 days before using them in experiments.

#### Detection of SARS-CoV-2 specific T cell lines

ELISpot assays were conducted using R5AB medium (RPMI 1640, 5% AB-Serum, 1% L-glutamine, 1% HEPES, 1% Pen/Strep). 96-well, nitrocellulose, filter-backed microtiter plates (Merck Millipore) were activated with 25μL 70% methanol per well and after washing twice with PBS, the plates were coated with 50μL of anti-human Interferon-γ antibody (Clone 1-D1K, Mabtech, Stockholm, Sweden) at a final concentration of 10μg/ml. After this, the plates were washed four times with PBS and blocked with R5AB. For the initial screening of SARS-CoV-2 stimulated cell culture, 50μL of the cell suspension each were added to 50μL R5AB per coated well. For further assays, cells were adjusted in R5AB medium to 1 × 10^5^ cells per 100μL in a total volume of 100μL per well. For analysis of freshly isolated PBMCs, 2 × 10^5^ cells were added in 100μL R5AB to the wells. Peptides were added directly to the wells at a final concentration of 20μg/ml. Cell suspensions without peptides served as negative controls. The plates were incubated for 20–36 h at 37°C and 5% CO_2_. After six washes with PBS containing 0.05% Tween 20 (PBS-T0.05%), 100μL biotinylated anti-human IFN-γ antibody (Clone 7-B6-1, Mabtech, Stockholm, Sweden) was incubated at a final concentration of 2μg/ml for 2 h at room temperature. After washing six times with PBS-T0.05%, 100μL avidin-peroxidase substrate (Vectastain Elite ABC-Kit, Linaris, Wertheim, Germany) was added to each well, the plates were incubated for one to 3 h at room temperature and then washed three times with PBS-T0.05% and three times with PBS. Finally, 100μL AEC substrate (3-amino-9-ehtylcarbazole, Sigma-Aldrich, Germany) activated with 0.06% H_2_O_2_ was added as a chromogen to each well. Spots developed within 10 min and the reaction was stopped by washing the plates three times with distilled water. The plates were airdried and the spots were counted using an ELISpot reader (AID, Strassberg, Germany). Results are reported as SFUs (Spot forming units) either per 50μL or per cell number, respectively. A peptide specific response was defined as positive if the number of SFUs exceeded the following thresholds: ≥10 SFUs and ≥2-fold over background (SFUs without peptide). For the assessment of the functional avidity of peptides by peptide titration assays, peptides were added in serial dilutions ranging from 20μg/ml to 1 ng/ml to ELISpot plates and incubated with T cell lines (1 × 10^5^ cells in duplicates) for 20-36h.

#### HLA typing and HLA-restriction analysis

HLA class I and II typing was performed using standard serological (Biotest AG, Germany) or different genotypical assays, including ELISA-based tests (enzyme linked probe hybridization assay Biotest ELPHA, Biotest AG, Germany), SSP (FluoGene ABC, Inno-Train, Germany) or SSO (LABtype, One Lambda, USA). Tests were carried out according to the standards of the European Federation for Immunogenetics (EFI) by an EFI accredited laboratory according to the manufacturer’s instructions. High resolution HLA typing was performed using either an SBT typing (Protrans HLA SBT S4, Protrans, Germany) or an NGS-based assay (MiaFora NGS MFlex, Immucor, USA). Tests were carried out according to the standards of the European Federation for Immunogenetics (EFI) by an EFI accredited laboratory according to the manufacturer’s instructions using CE-certified assays. SBT sequencing was performed on an ABI 3130 device (Applied Biosystems, USA) and analyzed using a CE-certified software (SBTengine, GenDx, Netherlands). NGS sequencing was performed on a MiSeq device (Illumina, USA) and analyzed using the test-associated software (MiaFora 5.2, Immucor, USA).

HLA-I/II restriction was demonstrated in ELISpot assays using antibodies blocking CD8 or HLA-I. The cells were incubated with a final concentration of 25μg/ml of an anti-CD8 (Clone SK1, BioLegend) or anti-HLA-I (Clone w6/32, BioLegend) antibody for 30 min at 37°C and 5% CO_2_. Then, peptides were added directly to the wells at a final concentration of 20μg/ml and the plates were incubated for 18–24 h at 37°C and 5% CO_2_.

The restricting HLA-I alleles of the epitopes were determined using B-LCLs from HLA-I typed individuals. Allogenic B-LCLs sharing ideally only one, but up to three HLA-I alleles with the T cell line were chosen, therefore restricting the potential presentation of the peptide to only this or these shared HLA-I alleles. These target cells were incubated with the respective peptides for 1 h at 37°C and washed three times with PBS. Peptide sensitized target cells and target cells without peptides were then co-incubated with the T cell line in an IFN-γ ELISpot assay. If not otherwise indicated, 5 × 10^4^ target cells and 1 × 10^5^ T cells were used in this assay and experiments were performed with technical duplicates.

#### HLA-B∗35 binding assay

For this assay, the HLA-B∗35:03^+^ Jurkat cell line (kindly provided by J. Dörrie, Department of Dermatology, University Hospital Erlangen) was used. 4 × 10^5^ cells were seeded in a 48 well plate in 250μL AIM-V medium. The desired peptide was added in a final concentration of 150μM, along with β2-microglobulin at a final concentration of 10 ng/ml to stabilize peptide-HLA complexes. The cells were incubated at 26°C and 7% CO_2_ for 18h followed by a 2h incubation at 37°C. Afterward, the cells were harvested and washed with FACS buffer (PBS, 0.5%BSA, 2mM EDTA) at 400xg for 5 min at room temperature. The samples were stained with mouse α-human B∗35 IgM in a final dilution of 1:40 for 15 min at room temperature. After a washing step with FACS buffer at 400xg for 5 min at room temperature, the cells were stained with goat α-mouse IgM-FITC in a final dilution of 1:200 for 15 min at room temperature. The samples were then washed with FACS buffer at 400xg for 5 min at room temperature and fixed with 4% PFA for 15 min at 4°C. After a final washing step with FACS buffer at 400xg for 5 min at room temperature, the cells were resuspended with FACS buffer and measured at the Navios Flow Cytometer. The data were analyzed with FlowJo v10.7.2 (BD Biosciences). Experiments were performed with technical unicates.

#### Fluorescence-activated cell sorting

1 × 10^7^ cells of the T cell line were stimulated with the respective peptide in a final concentration of 20μg/ml in R10IL2 for 3h at 37°C. Cells were then harvested and washed with cold MACS buffer (PBS, 0.5% BSA, 2mM EDTA) at 4°C before resuspending in 900μL cold R5AB medium.100μL Cytokine catch reagent (Miltenyi Biotec) were added and incubated for 5min on ice. Afterward, 10mL 37°C warm R5AB medium were added and cells were incubated for 45 min at 37°C. Samples were then put on ice and washed with cold buffer at 4°C. Afterward, cells were resuspended in 900μL cold MACS buffer, 100μL Cytokine detection antibody were added and incubated for 10min on ice. After washing with 10mL cold MACS buffer, the cells were incubated with α-CD8 BV421 at a final concentration of 0.2μg/ml (Clone SK1, BioLegend) and TotalSeq-C0258 (BioLegend) at a final concentration of 2.5μg/ml for 30min on ice. The samples were washed with 10mL cold MACS buffer and resuspended in sorting buffer (PBS, 0.5% BSA). Flow sorting of 12500 CD8^+^IFN-γ^+^ cells was carried out on a MoFlo Astrios EQ at the Core unit for cell sorting and immunomonitoring of the Nikolaus-Fiebiger-Center of Molecular Medicine (NFZ) Erlangen.

#### Single cell RNA sequencing and data analysis

Immediately after sorting, cells were loaded to a Chromium Next GEM Chip K (10X Genomics). Chromium Next GEM Single-Cell 5′ kits v.2 were used to generate VDJ libraries according to the manufacturer’s instructions (10X Genomics). Libraries were sent to Novogene and sequenced on an Illumina NovaSeq platform with PE150 strategy. 103.365.328 sequence read pairs were analyzed with the Single Cell V(D)J-Seq Analysis pipeline of CLC Genomics Workbench 23.0.3 (Qiagen Aarhus) to identify distinct TCR clonotypes. Briefly, the pipeline assembled reads from 11269 cell barcodes to 138976 contigs, which were the used to identify V, J, D and C segments. Cell annotations from Totalseq-C Hashtag C0258 sequences were created with the function Annotate Single Cell Reads and these were used to annotate the identified TCR chain clonotypes (6058).

#### HDR template design and production

DNA templates were designed in silico based on the previously published method^61^ and were synthesized by Twist Biosciences (San Francisco, California, USA) in pTwist Amp vectors. Briefly, the constructs comprise the full length of the a and b chains of the inserting TCRs flanked by left and right homology arms (LHA and RHA) and contain a poly-A tail (bGHpA), as well as self-cleaving peptides (P2A and T2A) to provide separation of a- and b-chain upon translation, as well as a poly-A tail (bGHpA). The b-chainTCRs consist of the human variable regions and the murine constant region, which is used as tracking marker. Linearized, double-stranded HDR templates were generated from the plasmids by PCR. Per 100μL PCR-reaction 60 ng plasmid DNA, 1μL Herculase II fusion polymerase (Agilent), 0.5 mM dNTPs, 0.4 μM HDR genomic forward and reverse primers, each, were added to 1x Herculase II Reaction Buffer. HDR templates were amplified in a PCR cycler (Thermofisher) using the following cycling conditions.

**Table:** 

Steps	Temperature	Time	Cycles
Initial Denaturation	95°C	3min	1
Denaturation	95°C	30s	34
Annealing	63°C	30s	
Extension	72°C	3min	
Final extension	72°C	3min	1
Hold	4°C	∞	

PCR products were purified with the Qiagen MinElute PCR purification Kit following the manufacturer’s instructions. Purified HDR templates were adjusted to a concentration of 0.5μg/μL for the electroporation. For the TCR constructs used in this study, see Table S6.

#### TCR re-expression and functional validation

TCR transgenic primary human T cells were generated by orthotopic TCR replacement as described in Moosmann et al.^61^ In brief, a T25 cell culture flask was coated at 4°C overnight with α-CD3 (BioLegend) and α-CD28 (BioLegend) in a final concentration of 1μg/ml in PBS. Freshly isolated PBMCs were adjusted to a concentration of 1 × 10^6^/mL in SC^+^ medium (RPMI 1640, 10% FBS, 5% SC^+^ supplement), supplemented with 300 U/ml IL-2 (peproTech), 5 ng/ml IL-7 (peproTech) and 5 ng/ml IL-15 (peproTech). The coated flask was washed twice with PBS before the cells were added and incubated for 48 h at 37°C and 5% CO_2_. 6h before the end of the incubation, half of the medium was removed and SC^+^ medium containing the cGAS-STING inhibitor RU.521 (Invivogen) was added to the cells in a final concentration of 4.82nM. After 6h of RU.521 treatment, cells were harvested, washed with R10 medium and adjusted to a concentration of 5 × 10^6^ cells/ml in SC^+^ medium. Mock cells directly transferred to a 24 well plate with pre-warmed SC- medium (RPMI 1640, 10% FBS, 5% SC^−^ supplement) at a density of 1 × 10^6^ cells/ml without electroporation. For each nucleofection, 1 × 10^6^ cells were used and seeded into a 96-well V-bottom plate. RNPs were generated in two sequential steps. First, the guide RNAs were prepared by mixing equimolar amounts of tracrRNA with hTRAC or hTRBC crRNA (final concentration 40μM) and heating at 95°C for 5min. After reaching room temperature, poly-L-glutamic acid (PGA, Sigma-Aldrich) was added to the hTRAC gRNA in a final concentration of 26μg/μL. In the second step, guide RNAs were assembled with Cas9 nuclease by mixing equal volumes of Cas9 nuclease (6 μM, IDT) and gRNA (40 μM) and incubating at RT for 15 min. An electroporation enhancer (IDT) was added to the RNP mixture in a final concentration of 20μM. P3 electroporation buffer (Lonza) was prepared as described by the manufacturer. Cells were spun down, resuspended in 20μL P3 electroporation buffer and mixed with 3.5μL hTRAC-RNP, 3.0μL hTRBC-RNP and 0.5μg HDR template. For KO-controls, only RNPs without HDR template were added. Cells were then electroporated with the EH100 electroporating setting on a Lonza 4D Nucleofector and immediately transferred to a 24 well plate with pre-warmed SC- medium at a density of 1 × 10^6^ cells/ml. After an incubation of 15 min at 37°C and 5% CO_2_, 0.05 μM Trichostatin A (Abmole) and 1 μM M3814 (Chemietek) were added to the PBMCs to increase the editing efficiency. After 24 h, the inhibitor containing medium was removed and replaced with fresh SC^+^ medium supplemented with 180 U/ml IL-2. 80-96h after electroporation, knock-in efficiency was validated by flow cytometry. Cells were washed with cold PBS+0.5% BSA and then stained with α-hTCR FITC (BioLegend, final 1:200), α-mTCR APC-Fire750 (BioLegend, final 1:100), α-CD8 eF450 (Life technologies, final 1:200), α-CD4 PE (Life technologies, final 1:400) and Zombie Aqua (BioLegend, final 1:200) in PBS+0.5% BSA. After an incubation period of 20min on ice, the cells were washed with cold PBS+0.5% BSA and then acquired on an LSR Fortessa flow cytometer. To validate the specificity of the transgenic TCRs, edited cells were incubated with B∗35:01 or B∗35:03 positive, peptide-loaded B-LCLs in an IFN-γ ELISpot assay.

#### Bioinformatic epitope prediction

Bioinformatic predictions of potential epitopes were carried out using the databases Syfpeithi^25^ and IEDB.^26^

### Quantification and statistical analysis

#### Statistical analysis

All statistical analysis was carried out using GraphPad Prism 9.0.2 (GraphPad Software Inc.). For unpaired datasets, two-tailed Mann-Whitney tests were used, for paired datasets two-tailed Wilcoxon matched-pairs signed rank tests were utilized. For analysis of contingency tables, two-tailed Fisher’s exact tests were used. Statistical detail of experiments can be found in the figure legends. Significance was defined as *p* ≤ 0.05.

## Supplemental information

Document S1. Figures S1–S6 and Tables S1–S3

Table S4. Characteristics of individuals included in stimulation experiments, related to STAR methods Excel file containing additional data too large to fit in a PDF.

Table S5. Characteristics of the 452 individuals included in the HLA analysis, related to STAR methods Excel file containing additional data too large to fit in a PDF.

Table S6. TCR constructs used in this study, related to STAR methods Excel file containing additional data too large to fit in a PDF.
