# Supplementary material for: Coronavirus replicase epitopes induce cross-reactive CD8 T cell responses in SARS-CoV-2-naive people with HIV-1
Source: iScience. 2025 Feb 3;28(3):111949. doi: 10.1016/j.isci.2025.111949 (PMC11872457; doi:10.1016/j.isci.2025.111949)
Supplement: Document S1. Figures S1–S6 and Tables S1–S3 [file mmc1.pdf]

iScience, Volume *28*

Supplemental information

Coronavirus replicase epitopes induce cross-reactive CD8 T cell responses in SARS-CoV-2-naive people with HIV-1

Katja G. Schmidt, Paulina Geißler, Ev-Marie Schuster, Christine Schülein, Ellen G. Harrer, Verena Schönau, Markus Luber, Bernd Spriewald, Philipp Steininger, Silke Bergmann, Armin Ensser, Kilian Schober, Krystelle Nganou-Makamdop, and Thomas Harrer

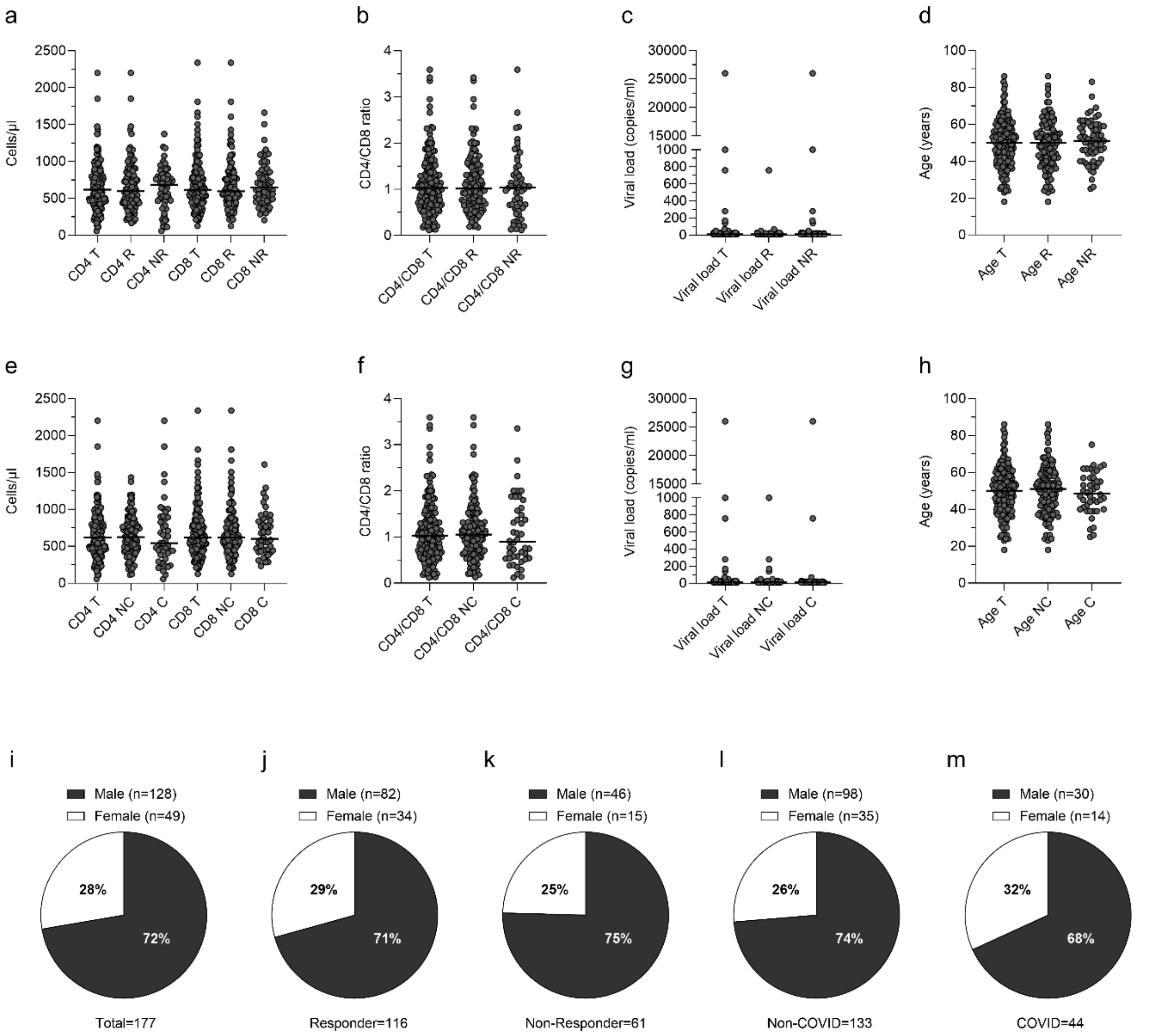

Figure S1: Characteristics of individuals included in the stimulation experiments. Presentation of **(a)** CD4 and CD8 T-cell counts, **(b)** CD4/CD8 ratio, **(c)** viral load and **(d)** age of responders and non-responders. Presentation of **(e)** CD4 and CD8 T-cell counts, **(f)** CD4/CD8 ratio, **(g)** viral load and **(h)** age of individuals with and without prior SARS-CoV-2 infection. Sex distribution within **(i)** all individuals, **(j)** responders, **(k)** non-responders, **(l)** individuals without prior SARS-CoV-2 infection, **(m)** individuals with prior SARS-CoV-2 infection. T: All 177 individuals included in the stimulation experiments, R: responders (n=116), NR: non-responders (n=61), NC, Non-COVID: individuals without prior SARS-CoV-2 infection (n=133), C, COVID: individuals with prior SARS-CoV-2 infection (n=44). FSC-A: Forward scatter area, FSC-H: Forward scatter height, SSC-A: Sideward scatter area. Related to STAR methods and Figure 1.

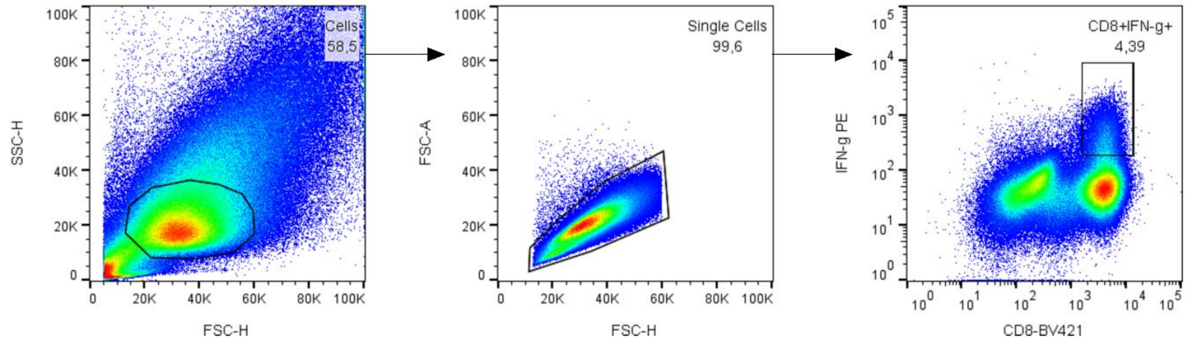

Figure S2: Gating Strategy for sorting of the CoV-YL8 specific T-cell line of donor #521. FSC-H: Forward scatter height, FSC-A: Forward scatter area, SSC-H: Sideward scatter height. Related to STAR methods and Figure 4.

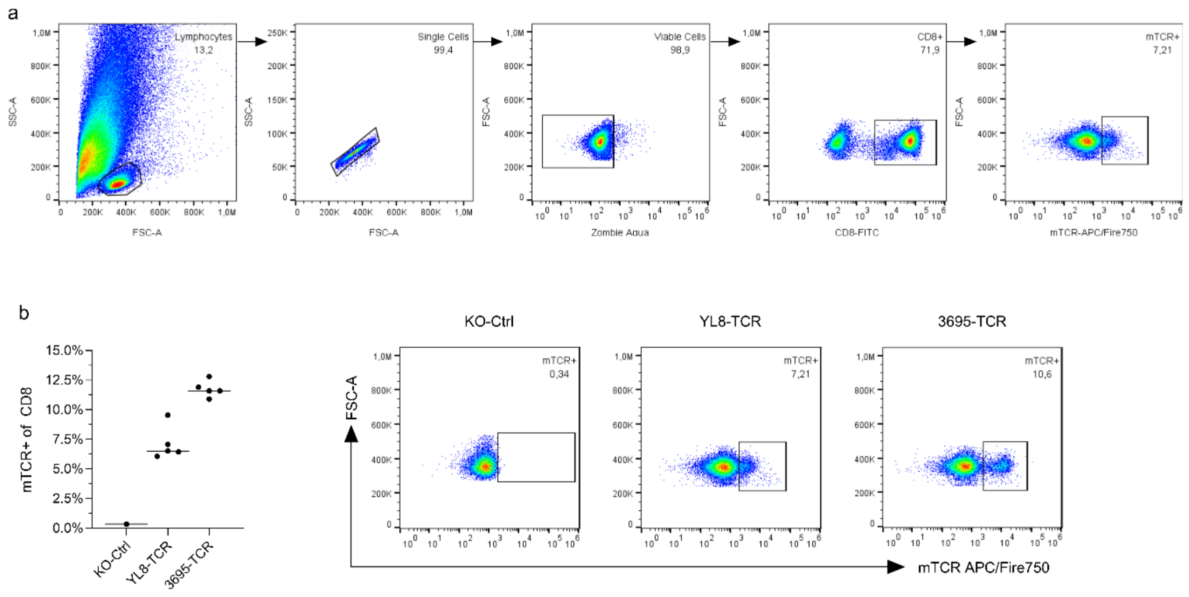

Figure S3: Expression of TCRs after CRISPR/Cas9-mediated editing. (a) Gating strategy for flow cytometric analysis of TCR expression. **(b)** Expression of transgenic mTCR β-chain in KO-Ctrl and the five electroporation replicates of YL8-TCR and 3695-TCR. Medians are indicated by the solid lines. **(c)** Expression of transgenic mTCR β-chain in KO-Ctrl, YL8-TCR and 3695-TCR after pooling of the electroporation replicates. FSC-H: Forward scatter height, FSC-A: Forward scatter area, SSC-H: Sideward scatter height. Related to STAR methods and Figure 4.

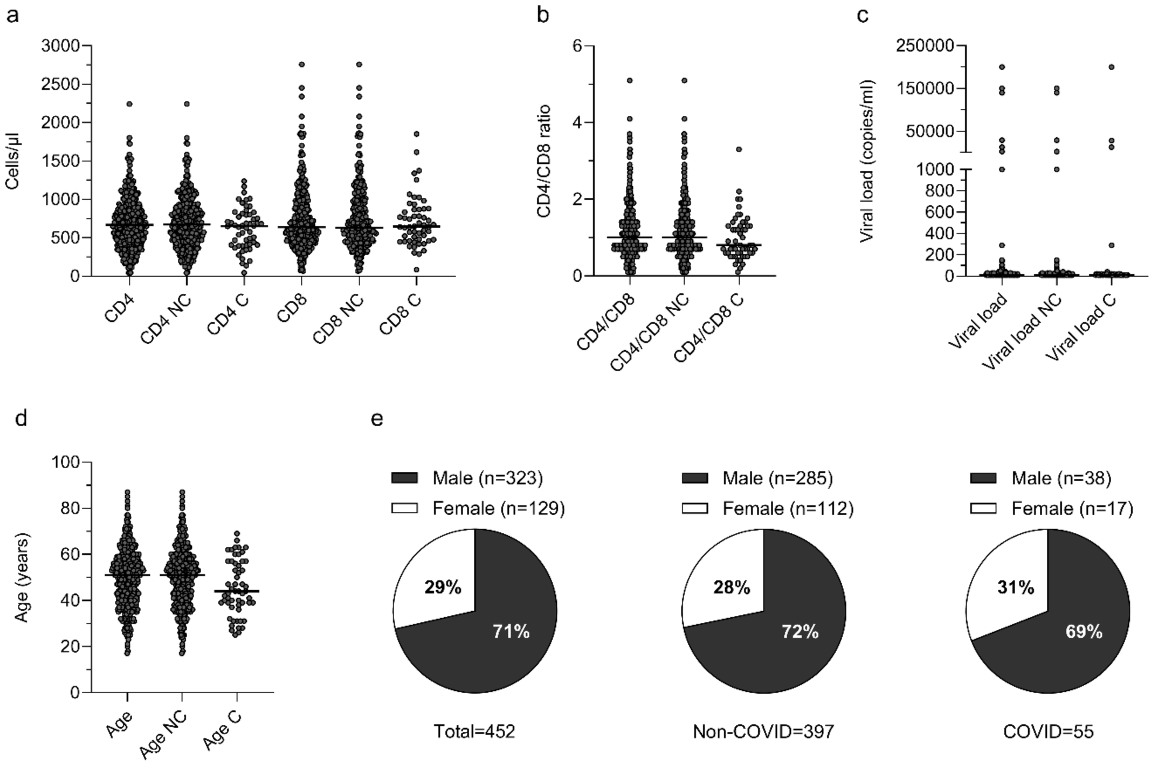

Figure S4: Characteristics of individuals included in the HLA analysis. Presentation of **(a)** CD4 and CD8 counts, **(b)** CD4/CD8 ratio, **(c)** viral load, **(d)** age and **(e)** sex distribution between individuals with and without prior SARS-CoV-2 infection. NC, Non-COVID: individuals without prior SARS-CoV-2 infection (n=397), C, COVID: individuals with prior SARS-CoV-2 infection (n=55). Related to STAR methods and Figure 5.

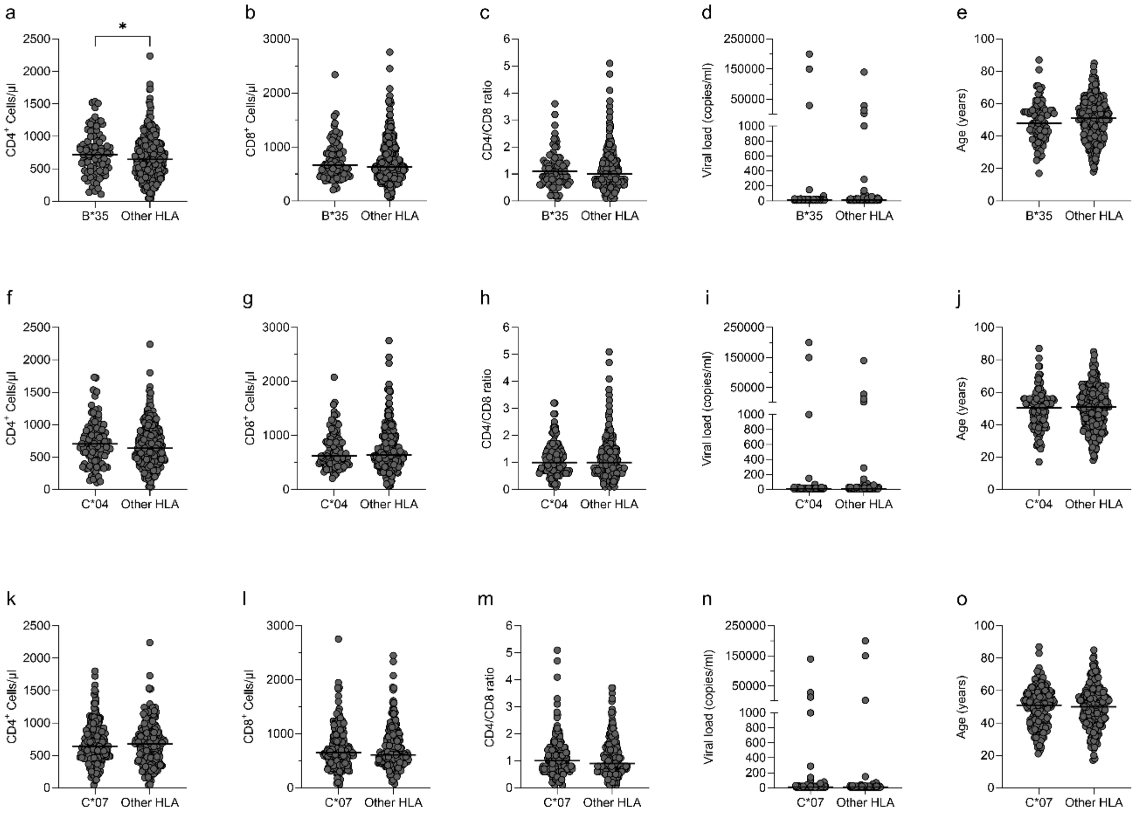

Figure S5: Characteristics of individuals included in the HLA analysis divided between HLA-I alleles. Presentation of **(a)** CD4 counts, **(b)** CD8 counts, **(c)** CD4/CD8 ratio, **(d)** viral load and **(e)** age between HLA-B*35 positive and negative individuals. Presentation of **(f)** CD4 counts, **(g)** CD8 counts, **(h)** CD4/CD8 ratio, **(i)** viral load and **(j)** age between HLA-C*04 positive and negative individuals. Presentation of **(k)** CD4 counts, **(l)** CD8 counts, **(m)** CD4/CD8 ratio, **(n)** viral load and **(o)** age between HLA-C*07 positive and negative individuals. Related to STAR methods and Figure 5.

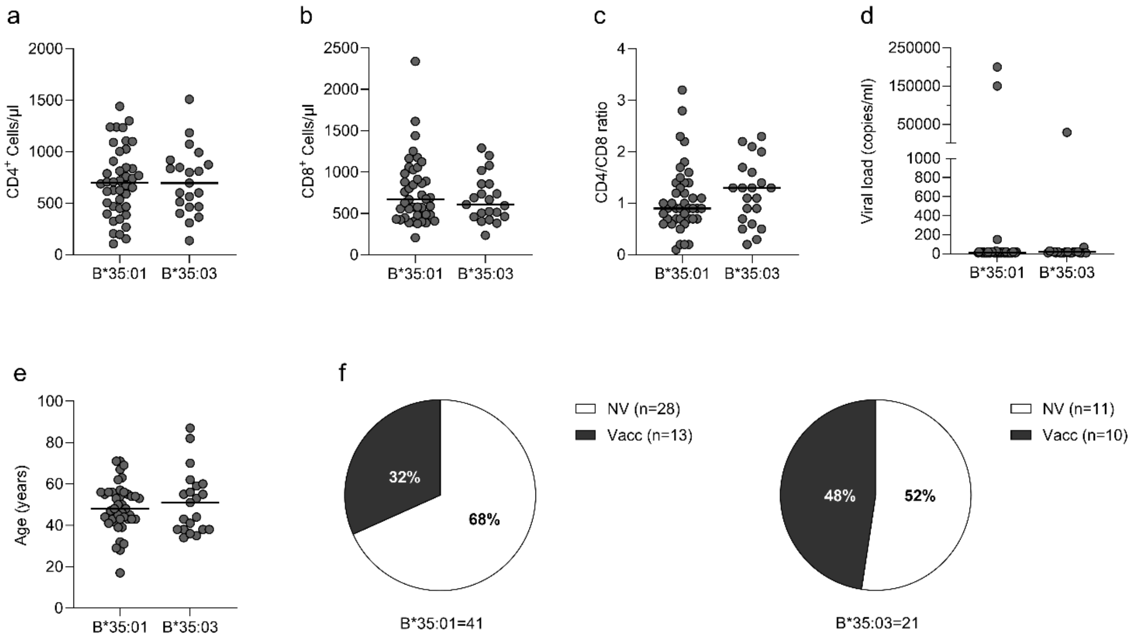

Figure S6: Characteristics of HLA-B*35:01 and HLA-B*35:03 positive individuals. Presentation of **(a)** CD4 counts, **(b)** CD8 counts, **(c)** CD4/CD8 ratio, **(d)** viral load, **(e)** age and **(f)** vaccination status distribution between HLA-B*35:01⁺ and HLA-B*35:03⁺ individuals. Related to STAR methods and Figure 5.

Table S1: Sequences of SARS-CoV-2/CCCoV peptides. Related to STAR methods and Figures 1, 2, 3 and 4.

Peptide	Pool	SARS-CoV-2 sequence	OC43 sequence	HKU1 sequence
RF16	1	RWVLNNDYYRSLPGVF	RWVLNNDYYRSLPGVF	SWVLNNDYYRSMPGTF
KL17	1	KVTSAMQTMLFTMLRKL	KVVSALQTMLFSMVRKL	KVVSALQTMLFSMVRKL
GY18	1	GIVGVLTLDNQDLNGNWY	GLVGVLTLDNQDLNGKWY	GLVGVLTLDNQDLYGQWY
DF14	1	DRCILHCANFNVLF	DRCIIHCANFNILF	DRCIIHCANFNILF
FA20	1	FAYTKRNVIPTITQMNLKYA	YAYTKRNVLPTLTQMNLKYA	YAYTKRNVLPTLTQMNLKYA
TV20	2	TITQMNLKYAISAKNRARTV	TLTQMNLKYAISAKNRARTV	TLTQMNLKYAISAKNRARTV
II15	2	ISAKNRARTVAGVSI	ISAKNRARTVAGVSI	ISAKNRARTVAGVSI
HI20	2	HLMGWDYPKCDRAMPNMLRI	VLMGWDYPKCDRAMPNILRI	VLMGWDYPKCDRAMPNILRI
HC20	2	HRFYRLANECAQVLSEMVMC	DRFYRLANECAQVLSEIVMC	DRFYRLANECAQVLSEIVMC
LS20	2	LYVKPGGTSSGDATTAYANS	YYVKPGGTSSGDATTAFANS	YYVKPGGTSSGDATTAFANS
GV18	3	GDATTAYANSVFNICQAV	GDATTAFANSVFNICQAV	GDATTAFANSVFNICQAV
SK15	3	SVLYYQNNVFMSEAK	QVLYYQNNVFMSESK	QVLYYQNNVFMSESK
VV22	3	VYLPYPDPSRILGAGCFVDDIV	VYLPYPNPSRILGAGCFVDDLL	VYLPYPDPSRILGAGCFVDDLL
GL18	3	GTLMIERFVSLAIDAYPL	SVLLIERFVSLAIDAYPL	SVLLIERFVSLAIDAYPL
TY21	3	TLQGPPGTGKSHFAIGLALYY	TVQGPPGTGKSHLAIGLAVFY	TVQGPPGTGKSHLAIGLAVYY
AL20	4	AKHYVYIGDPAQLPAPRTLL	AKHYVYIGDPAQLPAPRVLL	AKHYVYIGDPAQLPAPRVLL
CA20	4	CPAEIVDTVSALVYDNKLKA	CPKEIVDTVSALVYENKLKA	CPKEIVETVSALVYDNKLKA
NM20	4	NVNRFNVAITRAKVGILCIM	NVNRFNVAITRAKKGILCVM	NVNRFNVAITRAKKGIFCVM
RM11	4	RRLISMMGFKM	SRLISLMGFKL	SRLISLMGFKL
CV14	4	CVCSVIDLLLDDFV	SVCTVIDILLDDFV	SVCTVIDLLLDDFV
QI13	4	QKLALGGSVAIKI	DKLALGGSVAIKI	DKLSLGGSVAIKI
DF10		DYYRSLPGVF	DYYRSLPGVF	DYYRSMPGTF
YF9		YYRSLPGVF	YYRSLPGVF	YYRSMPGTF
SF10		SVLYYQNNVF	QVLYYQNNVF	QVLYYQNNVF
SV9		SVLYYQNNV	QVLYYQNNV	QVLYYQNNV
LPL10		LPYPDPSRIL	LPYPNPSRIL	LPYPDPSRIL
YL8		YPDPSRIL	YPNPSRIL	YPDPSRIL
CV9		CPAEIVDTV	CPKEIVDTV	CPKEIVETV
EV10		EIVDTVSALV	EIVDTVSALV	EIVETVSALV
DK13		DTVSALVYDNKLK	DTVSALVYENKLK	ETVSALVYDNKLK
DK11		DTVSALVYDNK	DTVSALVYENK	ETVSALVYDNK
NR9		NRFNVAITR	NRFNVAITR	NRFNVAITR
NV9		NVAITRAKV	NVAITRAKK	NVAITRAKK
AL10		AITRAKVGIL	AITRAKKGIL	AITRAKKGIF
IL9		ITRAKVGIL	ITRAKKGIL	ITRAKKGIF
RI9		RAKVGILCI	RAKKGILCV	RAKKGIFCV
YL9		YLQPRTFLL	PLTSRQYLL	PLSKRQYLL
LM9		LYYQNNVFM	LYYQNNVFM	LYYQNNVFM
YM8		YYQNNVFM	YYQNNVFM	YYQNNVFM

Amino acid substitutions between SARS-CoV-2 and OC43/HKU1 are marked in red. Pool: The peptides were contained in the indicated pool of peptides used for stimulation of PBMCs.

Table S2: Recognition of the peptide CoV-CV9 by patients with various B*35 subtypes. Related to Figure 2.

B*35 subtype	n patients	CV9		
		Responder	Median SFUs	Range SFUs
**B*35:01**	27	3	23	10-30
**B*35:02**	2	0	/	/
**B*35:03**	11	3	15	12-40
**B*35:05**	1	0	/	/
**B*35:06**	1	0	/	/
**B*35:01 and :03**	2	0	/	/
**N/A**	4	1	54	54
**Total**	48	7		

SFUs: Spot Forming Units, N/A; Not available.

Table S3: Number of patients with each HLA-I type. Related to Figure 5.

**HLA-I allele**	**n patients**		
	**Total**	**Non-COVID**	**COVID**
**A*01**	112	102	10
**A*02**	224	203	21
**A*03**	102	88	14
**A*11**	44	38	6
**A*23**	27	24	3
**A*24**	83	69	14
**A*25**	19	18	1
**A*26**	42	37	5
**A*29**	21	20	1
**A*30**	30	26	4
**A*31**	10	9	1
**A*32**	29	22	7
**A*33**	21	17	4
**A*34**	5	3	2
**A*36**	2	1	1
**A*43**	0	0	0
**A*66**	11	9	2
**A*68**	34	32	2
**A*69**	1	1	0
**A*74**	8	6	2
**A*80**	4	3	1
**B*07**	101	90	11
**B*08**	56	53	3
**B*13**	41	36	5
**B*14**	21	17	4
**B*15**	93	82	11
**B*18**	36	33	3
**B*27**	30	26	4
**B*35**	89	70	19
**B*37**	11	10	1
**B*38**	26	24	2
**B*39**	13	13	0
**B*40**	42	36	6
**B*41**	17	14	3
**B*42**	5	5	0
**B*44**	81	70	11

**B*45**	8	7	1
**B*46**	3	2	1
**B*47**	3	3	0
**B*48**	2	2	0
**B*49**	26	24	2
**B*50**	12	10	2
**B*51**	47	42	5
**B*52**	17	15	2
**B*53**	9	7	2
**B*54**	0	0	0
**B*55**	13	11	2
**B*56**	6	6	0
**B*57**	37	34	3
**B*58**	19	16	3
**B*59**	0	0	0
**B*67**	0	0	0
**B*73**	3	2	1
**B*78**	0	0	0
**B*81**	1	1	0
**B*82**	1	1	0
**B*83**	0	0	0
**C*01**	25	24	1
**C*02**	40	32	8
**C*03**	107	69	11
**C*04**	122	98	24
**C*05**	39	33	6
**C*06**	103	94	9
**C*07**	216	197	19
**C*08**	27	22	5
**C*12**	67	62	5
**C*14**	12	10	2
**C*15**	23	19	4
**C*16**	24	21	3
**C*17**	19	16	3
**C*18**	4	2	2
